# Biomolecule sulphation and novel methylations related to Guillain-Barré syndrome-associated *Campylobacter jejuni* serotype HS:19

**DOI:** 10.1099/mgen.0.000660

**Published:** 2021-11-01

**Authors:** Astrid P. Heikema, Nikolaos Strepis, Deborah Horst-Kreft, Steven Huynh, Aldert Zomer, David J. Kelly, Kerry K. Cooper, Craig T. Parker

**Affiliations:** ^1^​ Department of Medical Microbiology and Infectious Diseases, Erasmus University Medical Centre (Erasmus MC), Rotterdam, The Netherlands; ^2^​ Produce Safety and Microbiology Research Unit, Agricultural Research Service, United States Department of Agriculture, Albany, California, USA; ^3^​ Department of Infectious Diseases and Immunology, Faculty of Veterinary Medicine, Utrecht University, Utrecht, The Netherlands; ^4^​ Department of Molecular Biology and Biotechnology, University of Sheffield, Sheffield, UK; ^5^​ School of Animal and Comparative Biomedical Sciences, College of Agriculture and Life Sciences, University of Arizona, Tucson, Arizona, USA

**Keywords:** *Campylobacter jejuni*, Guillain-Barré syndrome, methylation, serotype HS:19, sulphation, whole-genome sequencing

## Abstract

*
Campylobacter jejuni* strains that produce sialylated lipooligosaccharides (LOS) can cause the immune-mediated disease Guillain-Barré syndrome (GBS). The risk of GBS after infection with *C. jejuni* Penner serotype HS:19 is estimated to be at least six times higher than the average risk. Aside from LOS biosynthesis genes, genomic characteristics that promote an increased risk for GBS following *C. jejuni* HS:19 infection, remain uncharacterized. We hypothesized that strains with the HS:19 serotype have unique genomic features that explain the increased risk for GBS. We performed genome sequencing, alignments, single nucleotide polymorphisms' analysis and methylome characterization on a subset, and pan-genome analysis on a large number of genomes to compare HS:19 with non-HS:19 *C. jejuni* genome sequences. Comparison of 36 *C. jejuni* HS:19 with 874 *C. jejuni* non-HS:19 genome sequences led to the identification of three single genes and ten clusters containing contiguous genes that were significantly associated with *C. jejuni* HS:19. One gene cluster of seven genes, localized downstream of the capsular biosynthesis locus, was related to sulphation of biomolecules. This cluster also encoded the campylobacter sialyl transferase Cst-I. Interestingly, sulphated bacterial biomolecules such as polysaccharides can promote immune responses and, therefore, (in the presence of sialic acid) may play a role in the development of GBS. Additional gene clusters included those involved in persistence-mediated pathogenicity and gene clusters involved in restriction-modification systems. Furthermore, characterization of methylomes of two HS:19 strains exhibited novel methylation patterns (5′-CATG-3 and 5′-^m6^AGTNNNNNNRTTG-3) that could differentially effect gene-expression patterns of *C. jejuni* HS:19 strains. Our study provides novel insight into specific genetic features and possible virulence factors of *C. jejuni* associated with the HS:19 serotype that may explain the increased risk of GBS.

## Data Summary

The authors confirm that all supporting data, and protocols have been provided within the article or through supplementary data files.

1. Whole-genome sequencing data generated in this study were made publically available and the accession numbers are provided at the end of this manuscript and in Table S1, available in the online version of this article. Accession numbers for other whole-genome sequencing data used in this study are provided in Methods.
2. Available metadata for *C. jejuni* HS:19 strains used in this study are provided in Table 1.
3. Supplementary tables, figures and data are available in the Supplementary Material file, deposited in figshare https://doi.org/10.6084/m9.figshare.14877510.v1.

**Impact Statement**

*
C. jejuni* strains with the HS:19 Penner serotype are highly associated with the development of Guillain-Barré syndrome (GBS), a rare post-infectious, antibody-mediated disease of the peripheral nerves. *C. jejuni* strains (serotype HS:19 and non-HS:19) that produce ganglioside-mimicking structures within their outer core lipo-oligosaccharides can cause GBS. It, however, is unclear why *C. jejuni* HS:19 strains impose an increased risk. In this study we performed comparative genomics to genetically characterized *C. jejuni* strains with the HS:19 serotype with the aim to determine if these strains have additional genetic features that may explain the increased risk of GBS. Genes and gene clusters unique for *C. jejuni* HS:19 strains, and novel methylation patterns were identified, further characterised and it was discussed how some of the identified gene clusters may contribute to GBS.

## Introduction

The foodborne pathogen *Campylobacter jejuni* is the worldwide leading cause of bacterial gastroenteritis. Besides diarrheal disease, an infection with *C. jejuni* can also result in the immune-mediated neuropathy Guillain-Barré syndrome (GBS). *C. jejuni* can have sialylated lipo-oligosaccharides (LOS) on its outer membrane [1, 2]. When induced during infections, antibodies to sialylated LOS may also bind to similar ganglioside structures present on peripheral nerves. The binding of such cross-reactive antibodies triggers complement-mediated immune activation with nerve damage and GBS as a result [3, 4].

The presence of ganglioside mimicking structures within the LOS is crucial for the induction of cross-reactive antibodies that recognize nerve gangliosides [5]. Furthermore, in an animal model, it was demonstrated that sialylated LOS purified from *C. jejuni* could induce the clinical symptoms that are observed in patients with GBS [6]. However, it should be noted that sialylated LOS by infecting *C. jejuni* alone is not sufficient in determining the post-infection development of GBS. Host and environmental factors likely play a role, and it is also possible that other bacterial virulence factors contribute to the development of GBS. Indeed, we previously demonstrated that six capsular genotypes are prevalent in *C. jejuni* strains isolated from patients with GBS [7]. Among these six capsular genotypes, the genotype HS19, corresponding to the Penner serotype HS:19, has been a focus of GBS-related research for decades. *C. jejuni* HS:19 strains are significantly associated with GBS in Japan, the USA and Bangladesh [7]. Japanese studies reported an overrepresentation (67 –75 %) of the HS:19 serotype in the GBS-related strains, compared to a low prevalence (6%) in *C. jejuni* strains from enteritis-only patients [8, 9]. Also, the risk of developing GBS after infection with *C. jejuni* HS:19 was estimated to be at least six times higher than with strains that have another serotype [10].

Extensive genetic diversity between *C. jejuni* strains is observed, but strains that have the HS:19 serotype are genetically conserved. Genetic typing methods such as restriction fragment length polymorphism and multilocus sequence typing (MLST) showed a close relationship [11] of the strains and a single dominance of the ST-22 clonal complex [7, 12]. Based on the increased risk of GBS and the close genetic relation of *C. jejuni* strains with the HS:19 serotype, we hypothesized that these strains have unique virulence factors that contribute to the development of GBS. In this study, we performed whole-genome sequencing of *C. jejuni* HS:19 strains. We addressed whether *C. jejuni* HS:19 strains isolated from patients with GBS could be distinguished from HS:19 strains isolated from patients with uncomplicated enteritis or from a food-related source. Additionally, using a large number of publicly available *C. jejuni* genomes, we searched for unique genes and gene clusters in *C. jejuni* HS:19 that could explain the increased risk of GBS.

## Methods

### Bacterial strains

Fifteen *C. jejuni* strains with the HS:19 serotype isolated from patients with GBS (*n*=8), uncomplicated enteritis (*n*=6) and a food source (*n*=1) were used in this study (see Table 1). Four GBS-related strains were isolated from the stools of Dutch patients. The other GBS-related strains were isolated from patients in Bonaire (*n*=1), the USA (*n*=3) or Japan (*n*=1). Five uncomplicated enteritis-related strains originate from Dutch patients, and one strain was isolated from the stool of a patient with enteritis from Canada. The complete genome sequence of this Canadian strain, also known as the HS:19 Penner reference strain, was published previously [13]. The food-related strain was isolated from packed chicken meat in the USA, and its full genome was also published previously [14]. The genomes of seven *C. jejuni* strains with serotypes other than HS:19 and genetically diverse backgrounds, other than ST-22, were also used in this study. This set of strains include NCTC 11168 (HS:2, ST-21, enteritis, accession no. AL111168), 81–176 (HS23/36, ST-42, enteritis, accession no. CP000538), 81 116 (HS:6, ST-283, enteritis, accession no. CP000814), RM1221 (HS:53, ST-354, poultry, accession no. CP000025), RM3196 (HS:41, ST-362, GBS, accession no. CP012690), GB19 (HS:4, ST-61, GBS, this study) and GB26 (HS:1, ST-21, GBS, this study) (Fig. S1). Strains were routinely cultured on Colombia blood agar plates (Becton Dickinson BV, Alphen aan den Rijn) containing 10 µg ml^−1^ vancomycin in a microaerophilic atmosphere at 37 ˚C.

**Table 1.** *
C. jejuni* strains with the HS:19 Penner serotype sequenced and used in this study. The LOS biosynthesis locus classes, capsular genotypes, MLST-ST and -CC were determined using PCR or blastn on whole-genome sequences. Mass-spectrometric analysis was used to determine the ganglioside mimics on the strains

Strain	Country isolated	Year isolated	Disease	Serotype	LOS class	Ganglioside mimic	MLST ST	MLST CC
GB03	The Netherlands	1995	GBS	HS:19	A1	GD1a,GM1a	22	ST-22
GB18	The Netherlands	1998	GBS	HS:19	A1	GD1a,GM1a	22	ST-22
GB28	Bonaire	1999	GBS	HS:19	A1	GD1a,GM1a	660	ST-22
GB60	The Netherlands	2015	GBS	HS:19	A1	nt	22	ST-22
RM1245	USA (California)	1996	GBS	HS:19	A1	nt	22	ST-22
RM1477	USA (Florida)	1983	GBS	HS:19	A1	nt	22	ST-22
RM1510	Japan		GBS	HS:19	A1	nt	22	ST-22
RM3147	Mexico		GBS	HS:19	A1	nt	22	ST-22
R12	The Netherlands	2002	Enteritis	HS:19	A1	GD1a,GM1a	22	ST-22
R23	The Netherlands	2002	Enteritis	HS:19	A1	GD1a,GM1a	22	ST-22
R31	The Netherlands	2002	Enteritis	HS:19	A1	GD1a,GM1a	22	ST-22
R72	The Netherlands	2002	Enteritis	HS:19	A1	nt	22	ST-22
R73	The Netherlands	2002	Enteritis	HS:19	A1	nt	22	ST-22
RM3420	Canada		Enteritis	HS:19	A1	nt	22	ST-22
RM1285	USA (California)	1997	non, chicken isolate	HS:19	A1	nt	22	ST-22

CC, clonal complex; MLST, multilocus sequence typing; NT, not tested; ST, sequence type.

### DNA isolation and whole-genome sequencing

Whole-genome sequencing was performed for the *C. jejuni* HS:19 strains in Table 1 and also the non-HS:19 strains GB19 and GB26. For the Dutch (includes strains GB19 and GB26) and Bonaire strains, genomic DNA was extracted from 2 days grown *C. jejuni* cultures using the DNeasy blood and tissue kit (Qiagen, Venlo, The Netherlands) according to the instructions of the manufacturer with the adjustment that vortexing and shaking were limited, the optional RNase treatment was included, and the DNA was eluted in 100 µl elution buffer AE (Qiagen, Venlo, The Netherlands). For strains RM1245, RM1477 and RM1510, genomic DNA was extracted from overnight grown cultures using sucrose-tris w/v phenol/chloroform clean-up protocol as described previously [15]. For all strains, whole-genome sequencing was performed using the Illumina MiSeq platform. Libraries were prepared using the KAPA LTP library preparation kit (Kapa Biosystems, Wilmington, MA). The pooled libraries were loaded into a MiSeq system and sequenced (depth >50X) using a MiSeq reagent kit version 2 with 2×250 cycles (Illumina). The reads for each genome were assembled the novo using the Newbler assembler (v2.6). For assembly refinement, an additional reference assembly against RM3420 was performed. Short read data are available on the NCBI SRA and are associated with the BioProject PRJNA634604.

Sequencing data generated were made publically available and the accession numbers are provided (Table S1). To assess DNA methylation patterns, sequencing was performed for strains RM1245 and RM1477, using Pacific Biosciences (PacBio, Menlo Park, CA) RSII with 20 kb SMRTbell libraries as described previously [16].

### *In silico* MLST

MLST was performed *in silico* by uploading the FASTA sequence of each genome in the Oxford PubMLST database for *Campylobacter* https://pubmlst.org/bigsdb?db=pubmlst_campylobacter_seqdef&page=sequenceQuery *,* or by using the MLST typing scheme for *C. jejuni *, of the Ridom SeqSphere+software (Ridom GmbH, Münster, Germany). Minimal spanning trees were constructed using BioNumerics software (v 7.6, Applied Maths NV, Sint-Martens-Latem) and Pearson correlations.

### Genome analysis

To assess the presence of large deletions, insertions or genomic rearrangements, whole-genome sequences of *C. jejuni* HS:19 strains were aligned using the Mauve plugin in Geneious (version R11, Biomatters, Auckland, New Zealand). For more in-depth analysis of the *C. jejuni* HS:19 genomes and the capsular- and LOS biosynthesis loci, the multiple muscle alignment plugin was used. Phylogenetic trees were constructed with the Geneious tree builder based on the Tamura-Nei distance model and the UPGMA tree build method.

### Gene presence-absence analysis

To identify unique genes within the genome of *C. jejuni* strains with the HS:19 serotype, a genome comparison based on a Roary pan-genome presence-absence analysis was performed. *C. jejuni* HS:19 strains mentioned in Table 1 and non-HS:19 strains with an unrelated genetic background (Fig. S1) mentioned in the Methods, were included in this analysis and strain RM3420 was used as a reference. Hereto, protein-, rRNA- and tRNA-encoding genes were identified using Prokka (v1.11) [17]. To determine the orthologous relationships of all proteins, protein sequences were clustered using Roary (v 3.8.1)[18] with an 85 % identity cut-off and with the paralogue splitting option disabled to prevent spurious orthologous gene cluster generation by genes located at contig breaks. The genomic regions specific for strains with the HS:19 serotype were visualized in a genomic map using the blast ring image generator (BRIG) [19]. To elucidate the biological function of hypothetical proteins, NCBI blastx was performed for each targeted gene, hits were aligned using Clustal Omega, and protein homologues were detected using HHpred and the PDB database (https://toolkit.tuebingen.mpg.de/tools/hhpred). SignalP (version 4.1) was used to assess the presence of signal sequences.

### Expanded gene presence-absence analysis

To further address the potential uniqueness of genes identified within the genome of *C. jejuni* HS:19, a second, severally expanded presence-absence analysis was executed. For this analysis, 1041 *C *. *jejuni* genomes were randomly obtained from the NCBI data repository using the shuf Linux command and the sub-set number 1041. The quality of the genomes was evaluated with CheckM (v1.1.2) using default parameters and the genome quality cut-off settings for inclusion: marker lineage=Campylobacter, completeness>=99 %, contamination<=1 %, number of contigs<=150, no filtering for heterogeneity). After this quality check, 895/1041 good-quality genomes remained and were used for further analysis. The genetic background (MLST) of the quality-checked genomes was determined using Ridom SeqSphere+ (Ridom GmbH, Münster, Germany), and LOS and capsule locus genotyping was done by local blastn analyses with LOS and capsule genotype-specific reference sequences. Of the 895 downloaded and quality-checked genomes, 21 were classified as HS:19 and 874 as non-HS:19. To determine if the downloaded genomes were a suitable representation of *C. jejuni* related to human infection, the MLST sequence types (MLST ST, seven genes) of the genomes were compared to MLST ST present in PubMLST, derived from strains isolated from human stools. For this comparison, a minimal spanning tree was constructed using BioNumerics software, and Pearson correlations were calculated.

Then, protein-, rRNA- and tRNA-encoding genes were identified using Prokka, and the orthologous relationships of all protein sequences were clustered using Roary (v3.12.0) with a 90 % identity cut-off. A Scoary (v1.6.16) [20] analysis was performed with the traits file for the analysis based on the genomes with the HS:19 serotype. Proteins significantly related to, or significantly absent in genomes derived from *C. jejuni* HS:19 strains (*P*-value<0.05, Benjamini–Hochberg corrected) were selected. The genes of these proteins and genes identified in the BRIG plot were subjected to an additional blastn analysis against all genomes. Genes with a blastn pairwise identity ≥80 % were included in further analysis.

blastn was also performed within PubMLST using a set of 287 genomes that all were ST-22 and had a core genome MLST (cgMLST) that had less than 200 cgMLST allelic differences according to *C. jejuni */* C. coli* cgMLST v1.0. The 287 genomics sequences were then queried within pubMLST using blastn against gene sequences (Data S1) derived from *C. jejuni* strain RM3420 and relevant in this study.

### Methylome analysis

The kinetic information contained in the PacBio SMRT sequencing data for strains RM1245 and RM1477 were utilized to characterize the methylome of these strains as described previously [21, 22]. Briefly, the methylomes of RM1245 and RM1477 that were sequenced and closed on the PacBio RSII were determined using the RS_Modification_and Motif_Analysis.1 protocol within the instruments SMRT Portal. The New England Biolabs Rebase tool (http://tools.neb.com/blast/) was used to identify the specificity of methyltransferases identified in *C. jejuni* HS:19.

## Results

### *C. jejuni* HS:19 strains have the same MLST type and a single LOS locus class

The genomes of 15 *C. jejuni* strains with the Penner serotype HS:19 were sequenced and further analysed. *In silico* MLST showed that all our sequenced HS:19 strains had a similar genetic background and belonged to ST-22 (Table 1). Previously, the LOS locus class of 14/15 of the strains used in this study were determined to be class A [1, 7, 23], while strain GB60 was newly determined for this study and also was class A. Intergenic variation that overlaps two genes (*cgtA* and *cgtB*) in the LOS class A biosynthesis locus led to the assignment of two separate alleles for LOS locus class A (A1 and A2) [24]. By aligning the sequence of the LOS loci to a known class A1 or A2 locus, we determined that the LOS loci of our sequenced strains all had an A1 locus (Table 1). All strains also had a shorter version of open reading frame three (orf3, *cj1035*) in the LOS biosynthesis locus with a missing protein domain of 98 amino acid residues. This shorter version encodes a one-domain glycosyltransferase that attaches a glycosyl group to HepI but not HepII, of the outer core LOS [24]. The absence of the glycosyl group on HepII allows the transfer of sialic acid to the adjacent galactose of the LOS outer core by campylobacter sialyltransferase (Cst-II) [24]. As a result, ganglioside mimicking structures, such as GM1a- and GD1a-mimics, can be produced. In agreement, these structures were detected when we performed mass-spectrometric analysis on purified LOS of six of our strains, GB3, GB18, GB28, R12, R23 and R31 [1, 25] (Table 1). GD1a and GM1a-mimicking structures are highly related to GBS but also produced by *C. jejuni* strains with other capsular serotypes than HS:19 [26].

### *C. jejuni* integrated elements are not related to GBS

To assess the presence of large genomic insertions, deletions or rearrangements, a Mauve alignment was performed on the sequenced genomes of the 15 *C *. *jejuni* HS:19 strains mentioned in Table 1. The Mauve alignment revealed the presence of~40 000 base-pair insertions at variable locations in the genomes of 6/15 strains (Fig. S2 and Table S2). The large insertions had 71–94 % query coverage, and 95.4–99.2 % sequence identity to *C. jejuni* integrated elements (CJIEs) reported in *C. jejuni* strain RM1221 [27]. Four of the HS:19 strains, GB3, RM1477, RM1510 and RM3420 possessed randomly inserted prophages similar to CJIE1, a Mu-like phage. HS:19 strain, R12, possessed CJIE2, which is a prophage that inserts at tRNA-Arg adjacent to *fusA* [28]. Another HS:19 strain, RM1285 had a unique prophage element integrated at the tRNA-Leu next to *atpE*. The presence of a CJIE in the genomes was not associated with GBS (*P*=1.000, GBS vs enteritis) and, CJIEs were not genetic markers for HS:19 strains. Aside from these CJIE insertions, the gene content of the genomes was generally syntenic, and rearrangements were not observed.

### Whole-genome and LOS loci sequence alignment did not result in a differentiation between GBS and enteritis

**Fig. 1.**
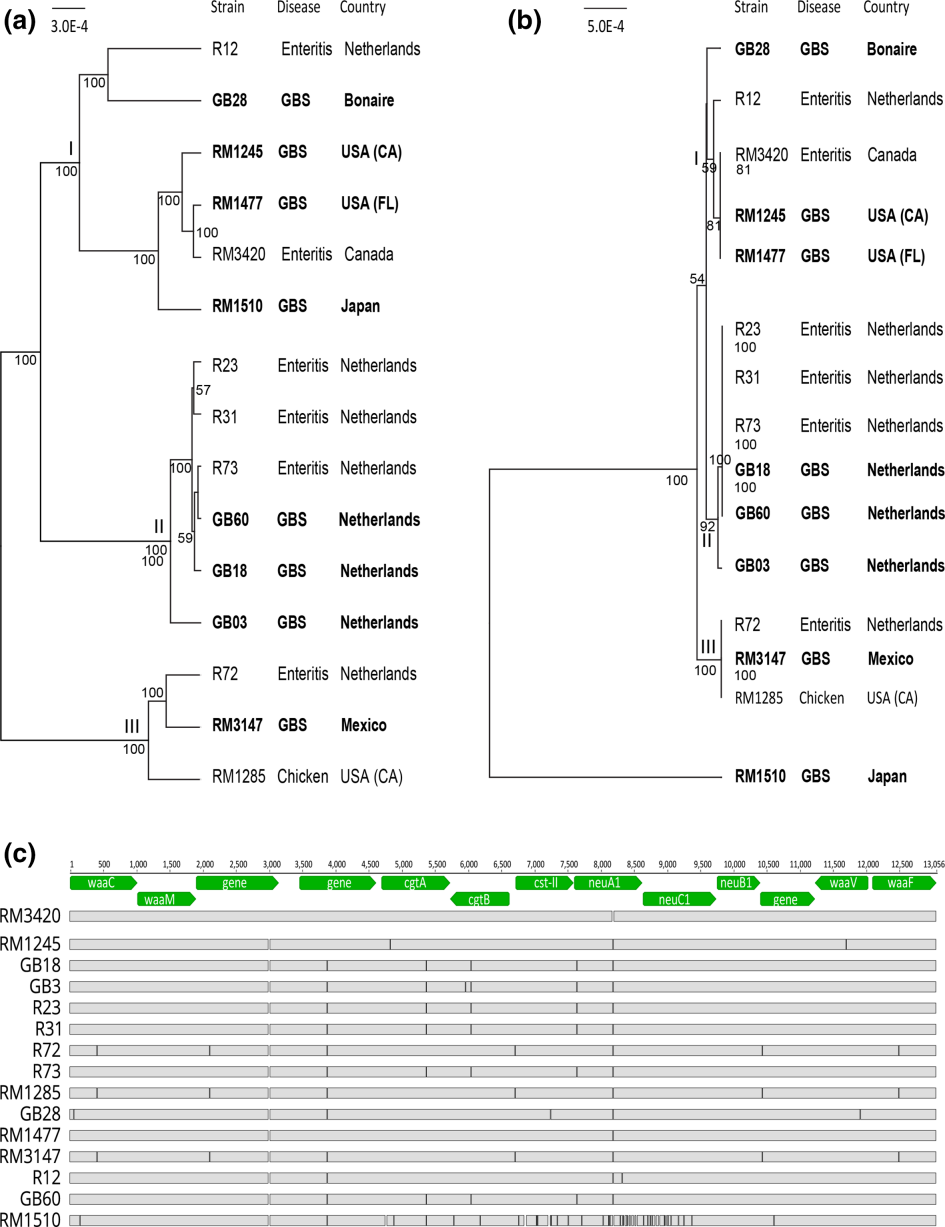
UPGMA tree of Mauve alignment complete genome sequences (a) and muscle-aligned LOS locus sequences (b). SNPs within the LOS biosynthesis locus (c).

To determine whether the genomes of GBS-related strains with the HS:19 serotype were generally different from enteritis-related strains with the HS:19 serotype, whole-genome alignments, using the muscle tool, were performed. For this analysis, the CJIEs were removed. Hierarchical Tamura-Nei/UPGMA clustering of the alignments showed that the strains were divided into three main clades (I-III) (Fig. 1a). Clade II contained Dutch strains only; in the other clades, strains originating from multiple countries, including from The Netherlands, were present. Importantly, GBS-related strains did not cluster away from the enteritis-related strains or the controls in any clade.

The genes present in the LOS biosynthesis locus determine the production of ganglioside mimicking structures that can trigger GBS. Strains sequenced in this study all have a LOS locus class A1 and typically express GM1a/GD1a-like structures (Table 1). Sequence variability within genes of LOS locus class A1 or the presence of mutations can lead to an altered LOS structure. To determine whether there are differences between the LOS biosynthesis loci of strains isolated from patients with GBS and strains isolated from patients with uncomplicated enteritis or a food source, a muscle alignment and UPGMA clustering was performed on the sequences of LOS locus genes *waaC-waaF* (Fig. 1b).

In general, only minor differences between the LOS loci sequences of GBS-, enteritis-related or the food strain were observed. In fact, two groups of strains (R72, RM1285, RM3147 and R23, R31, R73, GB18, GB60) had 100 % identical LOS locus sequences within the group and included enteritis-, a food strain as well as GBS-related strains. This indicates that the LOS locus sequence does not discriminate GBS- from enteritis-related or food-related strains.

An outlier in the phylogenetic tree (Fig. 1b) prepared from the LOS loci sequences was strain RM1510, derived from a Japanese patient with GBS. Compared to the RM3420 reference strain, this strain had 56 SNPs within the LOS biosynthesis locus (Fig. 1c), of which 25 led to an amino acid change (data not shown). The majority of SNPs in the LOS locus of strain RM1510 were present in three genes that encode proteins responsible for the transfer and synthesis of sialic acid: *cst-II* (*n*=15), *NeuB1* (*n*=18) and *NeuC1* (*n*=16) (Fig. 1c). Upon assessing the functionality of Cst-II, based on the amino acid at position 51 in the amino acid sequence, we found that strain RM1510 has an asparagine at this location (Ans51). Ans51 in Cst-II was shown to be critical for α2,8-sialyltransferase activity and a bifunctional Cst-II [29]. Bifunctionality of Cst-II in RM1510 implies that instead of GM1a- and GD1a-like structures, this strain can produce disialylated LOS structures. Subsequent blastn analysis of the sequence of genes *cst-II-NeuC1* of strain RM1510 showed a 100 % match with this region for *C. jejuni* strain OH4384 (HS:19), isolated from a patient with GBS in Canada. Mass spectrometry was performed on this strain, and in agreement with a bifunctional Cst-II, strain OH4384 produces disialylated GT1a-like LOS structures [30]. We, therefore, expect that our RM1510 strain also produces disialylated LOS structures. All the other strains had a mono-functional (Thr51) Cst-II.

The sequence of the capsule biosynthesis locus was highly conserved in all HS:19 strains. (Fig. S3a, b)

### Phase-variable genes were not a marker for GBS

As can be seen in Table S3, we identified 21 phase-variable genes that contain poly G/C-tracts in front, near the 5′ start, in the middle or near the 5′ end of the gene, in strains with the HS:19 serotype. For *C. jejuni *, phase variability is typically found in genomic regions involved in forming the flagella, capsule and the LOS. In agreement, we identified six genes with a poly G/C-tract involved in the biosynthesis and modification of the flagella (*CjjRM3420_1249, _1250, _1258, _1260, _1264, _1278*) and four in capsule modification (*CjjRM3420_0649, _1407, _1414, _1415*). Surprisingly, the LOS biosynthesis loci did not contain phase-variable genes. For the capsular gene *CjjRM3420_1407,* the poly G/C-tract was present in the upstream promotor region of the gene. Variation in length of such tracts can regulate gene transcription [31]. *CjjRM3420_1407* encodes the protein KpsS involved in intracellular biosynthesis of the capsular polysaccharide. Similar to KpsM mutants, KpsS mutants do not produce a capsule [32]. Changes in the length of the poly G/C-tract in the promotor region of CjjRM*3420_1407* may, therefore, orchestrate whether or not a capsule is present. In 2/15 the length of the poly-G/C-tract was 9 bp, in 12/15 it was between 9–11 bp and in 1/15 strains it was 12–13 bp. We did not assess how these differences may affect capsule production.

*CjjRM3420_1434*, encoding campylobacter sialyltransferase (Cst-I), located downstream of the capsule biosynthesis locus, has a poly-G/C tract in 11/15 strains, an interrupted (GGGGAGGGG) tract in 3/15, and no poly-G/C tract in 1/15 strains. This poly-G/C tract is located near the 5′ end of the gene and was always in a (partial) in-frame state. A terminal poly-G/C tract was also found in *CjjRM3420_1439* encoding a putative sulphotransferase. Such poly-G/C-tracts at the 5′ end of a gene are a means of gene-expression regulation [33]. Thus, a (frameshift) mutation in a poly-G/C-tract can lead to changes in transcription, or the expression of a truncated, not functional protein. We assessed whether variability in the poly-G/C tracts was a marker for GBS, but this was not the case (Table S3).

### Overrepresentation of genes and gene clusters in the genome of strains with the serotype HS:19

**Fig. 2.**
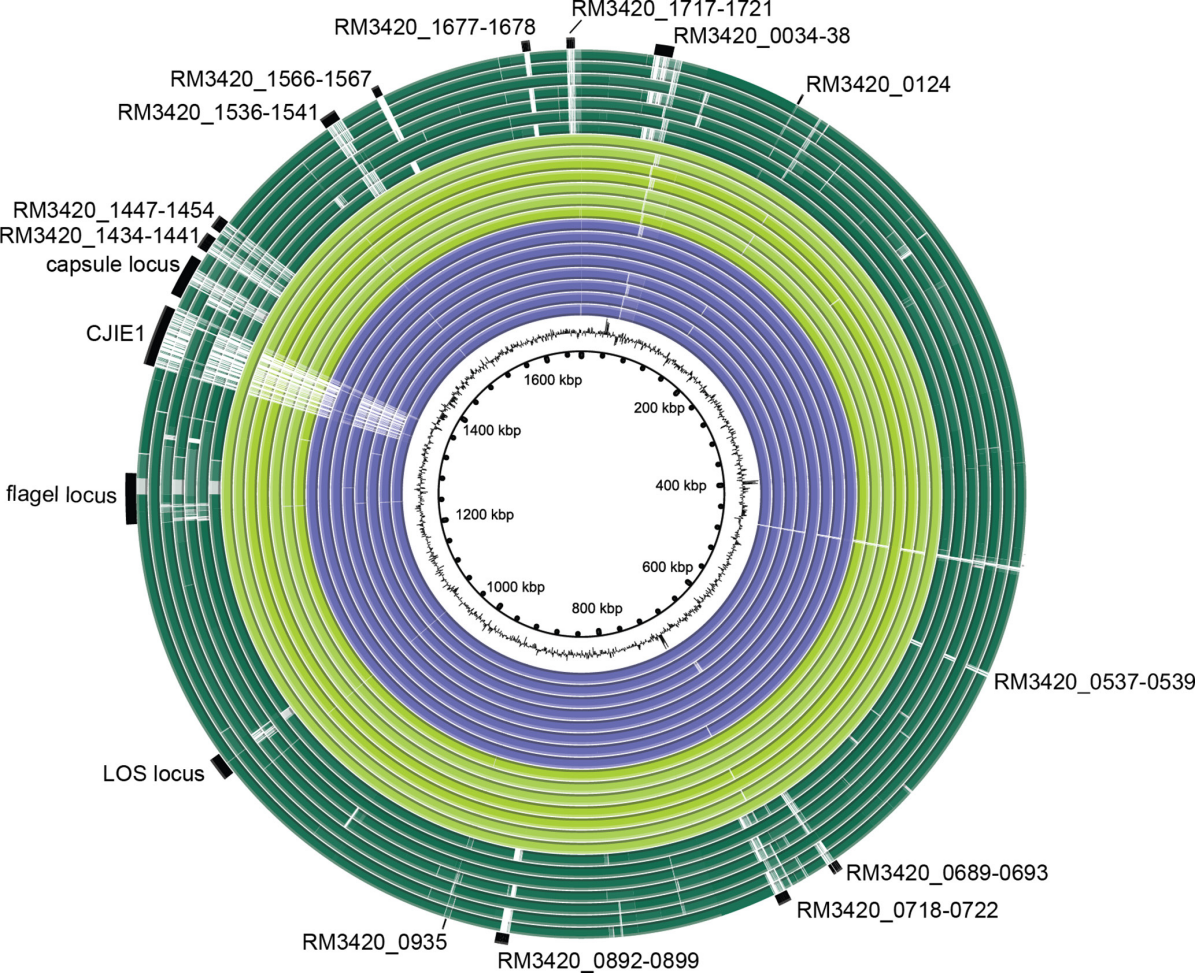
Genomic map with genomes of *C. jejuni* HS:19 and non-HS:19 strains. The blast ring image generator (BRIG) was used to indicate the location of relevant genes in a genomic map. Each genome is depicted as a coloured ring. The white areas indicate the absent genes when compared to the reference genome RM3420. The identity of the absent genes was addressed with a Roary pan-genome presence-absence analysis after Prokka annotation. Purple ring (inside to outside), GBS-related *C. jejuni* strains GB3, GB18, GB28, GB60, RM1245, RM1477, RM1510, RM3147; light green ring (inside to outside), enteritis-related *C. jejuni* strains R12, R23, R31, R72, R73, the chicken isolate RM1285 and the reference strain RM3420; dark green ring (inside to outside), non-HS:19 *C. jejuni* strains 11168 (HS:2), 81–176 (HS:23/36), 81116 (HS:6), RM1221 (HS:53), RM3196 (HS:41), GB19 (HS:4) and GB26 (HS:1).

To determine whether strains with the HS:19 serotype have unique genes including virulence factors that could explain the increased risk for GBS, Prokka annotation combined with a Roary pan-genome presence/absence analysis was performed. In an initial analysis, the genomes of strains listed in Table 1 and genomes of seven strains with a different serotype than HS:19 were included. The genomes of non-HS:19 strains comprised five previously published genomes; NCTC 11168, 81–176, 81116, RM1221 and RM3196, and two genomes of strains in our own collection: GB19 and GB26. The genomic regions specific for strains with the HS:19 serotype*,* including the gene regions that were absent when compared to the reference RM3420, were visualized in a genomic map (Fig. 2). Genes or clusters of sequential genes that were predominantly present in the HS:19 genomes, as identified by the Roary analysis and visualize by the BRIG plot, were indicated (Fig. 2).

With a presence of 15/15(100 %) in the genomes of strains with the HS:19 serotype and absence in 6/7(86 %) in the non-HS:19 genomes, the gene clusters *CjjRM3420_0689-0693, _0718-0722, _1434-1441, _1447-1454* and *_1717-1721* seemed most unique for strains with the HS:19 serotype. Genomic regions for the LOS and the flagella biosynthesis loci noted in the BRIG plot are shared by the genomes of HS:19 strains but have some genes absent from a few of the non-HS:19 genomes. Several genes within the capsule biosynthesis locus are unique for *C. jejuni* strains with the HS:19 serotype and were identified as absent in non-HS:19 genomes, as would be expected. The sequence of CJIE1, present in RM3420 and six of the other genomes, as mentioned above, was also clearly indicated in the BRIG plot, and an additional indicator of a reliable analysis.

### Genes and gene clusters significantly associated with *C. jejuni* HS:19 strains

To further address the uniqueness of these seemingly overrepresented genes and gene clusters within genomes of strains with the HS:19 serotype, an expanded Prokka annotation and Roary pan-genome presence/absence analysis was executed. Hereto, a large number (*n*=1041) of *C. jejuni* whole-genome sequences were randomly downloaded from NCBI. After CheckM verification, to assess the quality of the genome sequences, 895/1041(90 %) of the genomes passed the cut-off settings for inclusion. For these 895 genomes, MLST, capsule- and LOS locus genotyping was performed. There were 21 genomes classified as MLST CC ST-22, HS:19 and LOS locus class A1 and all HS:19 genotyped genomes were ST-22 (895–21=874 genomes were classified as non-HS:19). To determine if the genomes downloaded from NCBI were a good representation of *C. jejuni* genomes isolated from humans, the MLST ST of the downloaded genomes were compared to 10 000 randomly sampled MLST STs from PubMLST; visualized by a minimal spanning tree (Fig. S4). In the minimal spanning tree, the ST of the downloaded genomes from NCBI were evenly distributed over the PubMLST ST and correlated significantly (correlation MLST ST, NCBI vs PubMLST=0.771, *P*<0.001, Pearson, Fig. S4). The downloaded genomes from NCBI, therefore, were determined to be a good representation.

Then, Prokka annotation and the Roary pan-genome presence/absence analysis were executed on all *C. jejuni* HS:19 genomes (*n*=36, 15 were complete genomes, and 21 were obtained from NCBI as whole-genome shotgun contigs) and on the non-HS:19 genomes (only 25/874, 2.9 % were complete genomes), followed by a Scoary analysis to address associations with the HS:19 trait. The Prokka/Roary/Scoary analysis resulted in the identification of 91 protein coding sequences that were significantly present in or absent in *C. jejuni* HS:19 (*P*<0.05, with Benjamini–Hochberg correction for multiple testing). We, however, noticed that Roary identified more genes as present in the complete genomes compared to the whole-genome shotgun contigs. This, apparent, under-scoring seemed to be related to Prokka not annotating particular genes due to (non-random) contig breaks, truncation of genes due to mutations or out of frame poly G/C-tracts and undefined reasons. Prokka also had difficulties with differentiating proteins with similar functional domains including several glycosyltransferases and other transferases.

**Fig. 3.**
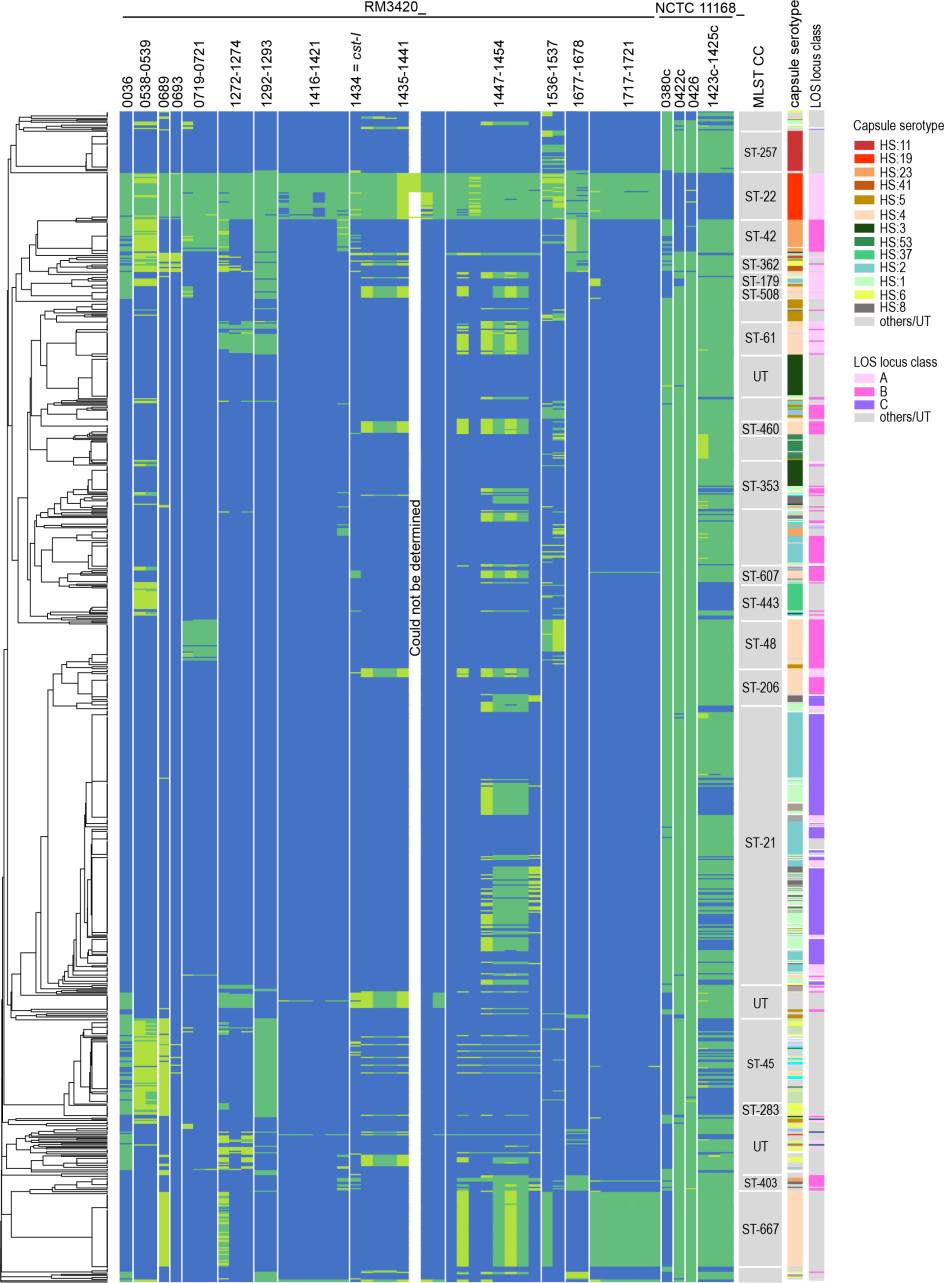
Presence and absence of genes significantly associated with *C. jejuni* Penner serotype HS:19. All genomes, *C. jejuni* HS:19 (*n*=36) and non-HS:19 (*n*=874), were annotated in Prokka and a Roary pan-genome analysis, together with Scoary statistics, was performed to determine the presence and absence of genes significantly associated with *C. jejuni* HS:19, depicted in a heatmap. The genomes in the heatmap were ordered based on hierarchical clustering of MLST sequence type data available for each genome (on the left). Each row in the heatmap represents a *C. jejuni* strain, each column a particular gene. The Penner serotype and LOS locus class (on the right) were determined by blast analyses with capsule serotype and LOS locus class-specific sequences. Green in the heatmap, the gene was identified as present by Roary and blastn; light green in the heatmap, the gene was identified as present by Roary or blastn; blue in the heatmap, the gene was identified as absent. Due to repeat-related contig breaks, CjjRM3420_1441 of cluster VI could only be detected in the completely assembled genomes.

We, therefore, subjected all genes identified by Scoary and/or visualize in the BRIG plot to additional blastn analysis to determine whether or not the gene indeed was present or absent. Eventually, 2179/46410 (4.7 %) of the Roary hits were corrected, with 91.5 % of the hits now being identified as present. After this correction, 51 genes remained being significantly associated with *C. jejuni* HS:19 (Fig. 3., Table 2, the CJIE genes were not included). Of these, six were identified as significantly absent in *C. jejuni* HS:19, and 45 genes were part of a cluster of two or more contiguous genes.

**Table 2.** Unique genes and gene clusters in *C. jejuni* strains with the HS:19 serotype. Genes and clusters of genes that were identified as significantly associated with *C. jejuni* HS:19 by comparing *C. jejuni* HS:19 (*n*=36) to *C. jejuni* non-HS:19 (*n*=874) genomes, after Prokka annotation, and by Roary pan-genome presence-absence analysis and Scoary statistics. The colours indicate the key (functional) categories. Green=restriction modification, blue=persistence, yellow=biomolecule sialylation and sulphation, grey=significantly absent in *C. jejuni* HS:19. ^a^Functionality predicted by HHpred and the PDB database (https://toolkit.tuebingen.mpg.de/tools/hhpred); ^b^A gene that contains a poly-G/C-tract

Annotation	Organization, ID	Product, E-value	Main function gene or cluster	Presence HS:19 (%, *n*=36)	Presence non-HS:19 (%, *n*=874)	Presence MLSTs / dominant serotypes
CjjRM3420_0036	single gene	thioredoxin-related protein_SoxW family	sulphur metabolism	97	17	ST-42/HS:23, ST-362/H:41, ST-179/HS:4, ST-508/HS:4, ST-45/mix, ST-283/HS:6, ST?/HS:6
CjjRM3420_0538	cluster I	hypothetical protein	unknown	100	19	ST-42/HS:23, ST-362/HS:41, ST-179/mix, ST443/HS:37, ST-45/mix, ST-283/HS:6
CjjRM3420_0539		hypothetical protein		100	19	
CjjRM3420_0689^a^	single gene	hypothetical protein	–	97	8	ST-362/H:41, ST-21/HS:1–2
CjjRM3420_0693	single gene	metallo-endopeptidase, E 8.1e-23		97	19	ST-362/H:41, ST-45/mix ST667/HS:4
CjjRM3420_0719		putative type I restriction enzyme	restriction modification	97	8	ST-42/HS:23, ST-48/HS:4
CjjRM3420_0720	cluster II	hypothetical protein		100	6	
CjjRM3420_0721		putative ATP-dependent endonuclease		100	6	
CjjRM3420_1272		motility associated factor, glycosyltransferase family	flagel assembly	94	18	ST-61/HS:4, ST?/mix, ST677/HS:4
CjjRM3420_1273	cluster III	motility associated factor, glycosyltransferase family		100	5	
CjjRM3420_1274		motility associated factor glycosyltransferase family		100	7	
CjjRM3420_1292^a^	cluster IV	toxin-antitoxin complex, mRNA interferase, E 1.2e-6	toxin-antitoxin / translation control	100	20	ST-42/HS:23, ST-362/HS:41, ST-179/HS:4, ST-508/HS:4, ST677/HS:4, ST-61/HS:4, ST-45/mix
CjjRM3420_1293		type II toxin-antitoxin system, RelE/ParE family toxin		97	20	
CjjRM3420_1416	cluster V	putative glycosyltransferase	capsule biosynthesis	67	0	
CjjRM3420_1417		putative phosphatase		97	0	
CjjRM3420_1418		putative aminotransferase		97	0	
CjjRM3420_1419		galactosylsyltransferase		58	0	
CjjRM3420_1420, ugd		UDP-glucose 6-dehydrogenase		100	0	
CjjRM3420_1421		putative glycosyltransferase involved in capsule biosynthesis		94	5	ST-42/HS:23
CjjRM3420_1434, cstI^b^	cluster VI	alpha-2,3-sialyltransferase I		100	5	ST-607/H:S4, ST-403/HS:23-mix, radom
CjjRM3420_1435, cysD		sulphate adenylyltransferase subunit 2		100	8	ST-508/HS:4, ST-460/HS:4, ST-206/HS:4, ST-1435/HS: 1437
CjjRM3420_1436, cysN		sulphate adenylyltransferase subunit 1	sialylation / sulphation	100	8	
CjjRM3420_1437^a^		anion permase, putative sodium/sulphate cotransporter, E 2.8e-24		100	8	
CjjRM3420_1438, cysC		adenylylsulphate kinase		100	8	
CjjRM3420_1439^b^		putative sulphotransferase		nd	nd	nd
CjjRM3420_1440		glycosyltransferase		92	0	–
CjjRM3420_1441^a, b^		anaerobic sulphatase-maturating enzyme, E 4e-10		100	2	ST-?/HS:?
CjjRM3420_1447	cluster VII	hypothetical protein	methylation / capsule biosynthesis	100	0	–
CjjRM3420_1448^b^		methyltransferase		92	15	ST-179/HS:4, ST-508/HS:4, ST-61/HS:4, ST-460/HS:4, ST206/HS:4, ST-677/HS:4
CjjRM3420_1449^a^		radical SAM metalloprotein, E 6.0e-28		100	1	
CjjRM3420_1450^a^		hypothetical protein with SnoaL-like domain, E 8.5e-18		100	20	ST-179/HS:4, ST-61/HS:4, ST-460/HS:4, ST-206/HS:4, ST-21/HS:1
CjjRM3420_1451		putative glycosyltransferase		100	34	ST-179/HS:4, ST-508/HS:4, ST61/HS:4, ST460/HS:4, ST-206/HS:4, ST-21/HS:1, ST-677/HS:4
CjjRM3420_1452		potentually involved in capsule biosynthesis		97	33	
CjjRM3420_1453		capsular polysaccharide biosynthesis protein		100	34	
CjjRM3420_1454		Sam-dependent methyltransferase		97	6	ST-21/HS:8
CjjRM3420_1536, rloF	cluster IIX	restriction modification linked orf	restriction modification	97	16	ST-257/HS:11, ST-574/HS:mix, ST:48/HS:4, ST-677/HS:4
CjjRM3420_1537, hsdS		type I restriction-modification system, specificity subunit S		100	12	ST-257/HS:11, ST-574/HS:mix, ST:48/HS:4
CjjRM3420_1677	cluster IX	dipeptidyl peptidase		94	7	ST-42/HS:23, ST-362/HS:41
CjjRM3420_1678^a^		membrane transport protein, E 7.7e-28		81	7	ST-403/HS:mix
CjjRM3420_1717	cluster X	pseudogene	restriction modification	100	8	ST-677/HS:4
CjjRM3420_1718		DNA methyltransferase		100	7	
CjjRM3420_1719		hypothetical protein		100	7	
CjjRM3420_1720		hypothetical protein, contains a zinc-ribbon domain		97	7	
CjjRM3420_1721		restriction endonuclease		97	7	
CjjRM3420_1722^a^		sensor protein/putative methyltransferase, E 9.6e-15		100	7	
Cjj11168_0380c	single gene	hypothetical protein		0	73	ST-179/HS:4, ST-508/HS:4, ST-45/HS:mix, ST-677/HS:4
Cjj11168_0422c	single gene	putative H-T-H containing protein		0	94	ST-42/HS:23
Cjj11168_0426	single gene	putative ABC transporter ATP-binding protein		6	96	ST-42/HS:23
Cjj11168_1423c		d-glycero-alpha-d-manno-heptose 1-phosphate guanylyltransferase	capsule biosynthesis non-HS:19 capsule	0	79	ST-443/HS:37, ST-21/HS:1, ST-45/HS:mix, ST-283/HS:6
Cjj11168_1424c	cluster IX	Phosphoheptose isomerase		0	79	
Cjj11168_1425c		d-glycero-alpha-d-manno-heptose 7-phosphate kinase		0	79	

NA, not applicable; UT, untypable.

### Sulphur metabolism, sulphur modification, restriction-modification systems and virulence factors and related to *C. jejuni* HS:19

To further investigate the function of the genes and clusters of genes (indicated as clusters I–XI, Table 2) that were significantly associated with *C. jejuni* HS:19, HHpred annotation was performed on genes (*n*=18) encoding hypothetical proteins. A function could be assigned to eight of these genes (Table 2).

Then, we aimed to further study the genes and gene clusters to determine if they could contribute to the increased risk of developing GBS. Of the genes and clusters identified, gene *CjjRM3420_0036,* encodes a thioredoxin-related protein of the SoxW family. In various bacteria, this gene is strongly associated with a *mcc* cluster of genes involved in dissimilatory sulphite reduction [34] in which inorganic sulphite is reduced to sulphide. Homologues of these genes are present in *C. jejuni* HS:19 (*CjjRM3420_0034-0037),* but the Roary/Scoary analysis only identified *CjjRM3420_0036* as being associated with *C. jejuni* HS:19. The genome alignments, visualized with at BRIG plot (Fig. 2), and subsequent blastn analysis did, however, show that genes *CjjRM3420_0034, _0035 and _0037* were *C. jejuni* HS:19-related as well (97 % in HS:19 vs 16–17 % in non-HS:19 strains). The Roary/Scoary analysis appeared to have missed these genes due to the presence of several point mutations in the *mcc*-like gene cluster, including a mutation that leads to a truncated dissimilatory sulphite reductase (*CjjRM3420_0034*). This reductase plays a key role in dissimilatory sulphite reduction but misses an N-terminal signal sequence in *C. jejuni* HS:19 that is critical for its periplasmic localization and function. In agreement, gene *CjjRM3420_0034* was annotated as a pseudogene in strain RM3420 [13]. Based on the presence of multiple mutations, we concluded that the *mcc*-like cluster needed for sulphite reduction is probably not functional in *C. jejuni* HS:19 strains. The physiological function of a self-contained thioredoxin-related protein is currently unknown.

Three gene clusters: II (*CjjRM3420_0719-0722*), IIX (*CjjRM3420_1536-1537*) and X (*CjjRM3420_1717-*1722), (Table 2, indicated in green), have genes that encode endonucleases and methyltransferases. These enzymes are often part of restriction-modification systems that protect bacteria from the integration of foreign DNA. To determine whether HS:19 strains can take up foreign DNA, natural transformation assays were performed as describes [35] using donor DNA derived from a *C. jejuni* knockout strain GB11Δ*cstII* (HS:2 [5]) or 11168Δ*kpsM*. Most strains (10/15) were transformable except for the four strains that contained a CJIE1 and strain RM1245 (Table S2). Apparently, the endonucleases and methyltransferases encoded in clusters II, IIX and X do not inhibit the uptake and integration of chromosomal DNA in 10/15 *C. jejuni* HS:19 strains, at least not when the DNA is derived from other (n=2) *C. jejuni* strains.

When secreted, endonucleases can enhance pathogenicity, for example, by destroying antimicrobial neutrophil extracellular traps (NETs) that are partly composed of DNA [36]. To investigate whether the identified endonucleases in the genomes of *C. jejuni* HS:19 were secreted in the environment, a screening for signal sequences that allow the translocation of the protein across the membrane was performed. Using SignalP 4.1, we determined that none of the nucleases had a signal sequence for extracellular sercretion. In summary, our findings suggest that the identified endonucleases are not secreted in the environment, but together with the methyltransferases, probably protect the bacterium from the integration of foreign DNA derived from different bacterial species or other sources.

Cluster III contains flagella-associated genes (*CjjRM3420_1272-1274*). These genes are conserved in *C. jejuni* but showed allelic diversity compared to other capsular serotypes, with pairwise identities that generally were below our cut-off of 80 % (at the DNA level).

Genes in the small cluster IV (*CjjRM3420_1292-1293,* Table 2, indicated in blue) encode a type II toxin-antitoxin system of the RelE/ParE system, of which *CjjRM3420_1292* encodes the toxin and *_1293* the antitoxin. This toxin-antitoxin system has a regulatory function and enables bacteria to persist under unfavourable conditions in a dormant state [37, 38].

An intriguing feature of *C. jejuni* HS:19 is that it has two sialyltransferases. Besides the LOS locus class A- and B-specific sialyltransferase gene *cst-II*, *C. jejuni* HS:19 also have the campylobacter sialyltransferase gene *cst-I* (*CjjRM3420_1434*). This gene is the first gene of cluster VI (Table 2, indicated in yellow) and lies in close proximity to the capsule biosynthesis locus. Sialylation of LOS by Cst-II is crucial for the production of anti-ganglioside antibodies [5] and the main virulence factor of GBS [6]. To determine whether Cst-I is also able to sialylate the LOS, a *cst-II* knockout mutant was generated in *C. jejuni* HS:19 strain GB18. In contrast to the GB18 wild-type strain, the *cst-II* knockout mutant was unable to bind to cholera toxin or sialoadhesin, proteins that specifically bind to sialylated structures such as GM1 and GD1a (Fig. S5) [39]. Cst-I, therefore, does not seem to sialylate the LOS leading to the production of the ganglioside-mimicking structures GM1 and GD1a, present in the wild-type GB18 (Table 1).

**Fig. 4.**
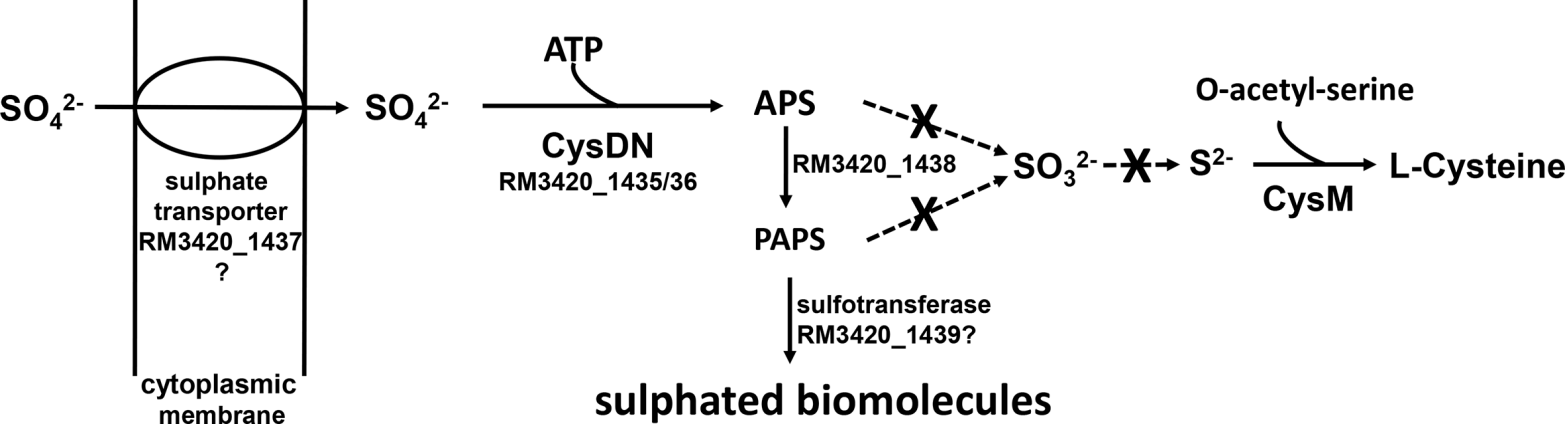
The proposed function of cluster VI. Sulphate is transported into the bacterial cell by a sulphate transporter (*CjjRM3420_1437*). Next, the sulphate is activated by an ATP sulphurylase (*CjjRM3420_1435/36*), leading to the formation of adenosine 5'-phosphosulphate (APS). Then, an APS kinase (*CjjRM3420_1438*) catalyses the formation of 3'-phosphoadenylyl sulphate (PAPS). PAPS is a substrate for sulphotransferase (*CjjRM3420_1439*) that can add sulphate to biomolecules such as proteins and carbohydrates.

*CjjRM3420_1435* and *_1436* of cluster VI (Table 2, indicated in yellow), encode CysD and CysN homologues that are the two subunits, which form sulphate adenylyltransferase. In several other bacteria, sulphate adenylyltransferase is involved in the assimilation of sulphate for biosynthesis of cysteine. Nevertheless, the *CjjRM3420_1435* and *_1436* encoded putative sulphate adenylyltransferase involvement in cysteine biosynthesis is unlikely as it cannot perform assimilatory sulphate reduction due to the absence of CysG, CysH and CysJ, which are crucial enzymes of this pathway [34, 40]. Instead, *C. jejuni* relies either on an exogenous cysteine supply or else sulphide/thiosulphate to make cysteine from O-acetylserine via CysM [41]. However, this putative sulphate adenylyltransferase could have an alternative function as it can also work together with proteins encoded by genes *CjjRM3420_1438* and *_1439* towards modifying proteins and carbohydrates with sulphate. Sulphate adenylyltransferase catalysis the reaction ATP+sulphate ⇌ PPi+adenosine 5'-phosphosulphate (APS, Fig. 4). Then, the *CjjRM3420_1438* gene-encoded adenylylsulphate kinase catalyses the reaction ATP+APS ⇌ ADP+3'-phosphoadenylyl sulphate (PAPS). PAPS is a substrate for sulphotransferases (*CjjRM3420_1439* encoding a putative sulphotransferase) that transfers sulphate to an alcohol (OH-) or amine (NH-) group. Such alcohol- and amine-groups are present as modifications on proteins and carbohydrates, including the LOS and the capsule. *CjjRM3420_1437* of cluster VI (Table 2, indicated in yellow) encodes a putative sulphate transporter that could enable bacterial uptake of inorganic sulphur for this purpose. Finally, the putative anaerobic sulphatase-maturating enzyme encoded by *CjjRM3420_1441*, is a radical SAM enzyme that catalyses the activation of sulphatases. These enzymes can, for example, desulphate mucins under anaerobic conditions [42]. Thus, this cluster VI (Table 2, indicated in yellow) seems to encode a set of proteins that we hypothesize allows the uptake of sulphate and its conversion to PAPS, which can then act as an activated sulphate donor to covalently modify key biomolecules, which might be important in virulence.

It should be noted that three of the unique genes in cluster VI (Table 2, indicated in yellow), including *cst-I* and the anaerobic sulphatase-maturating enzyme, contain homopolymeric G/C-tracts (Tables 2 and S2). The G/C-tracts mediate reversible on/off switching of genes and can provide advantages to bacteria in a specific niche. Further future experiments are needed to assess if these homopolymeric G/C-tracts can be linked to virulence and GBS.

Overall, the associations between the identified gene clusters and *C. jejuni* HS:19 were further confirmed using blastn analysis within PubMLST using a set of 287 ST-22 *C. jejuni* strains. All ST-22 strains possessed LOS class A1 and the HS:19 specific capsule locus sequence (Table S4). Greater than 95 % of the ST-22 strains from PubMLST possessed HS:19 clusters I, II, III, IV, VI and X based on>98 % identity and >60 % query coverage.

### Co-occurrence of the identified cluster in non-HS:19 *C. jejuni* genomes

Despite a significant association, several genes and (parts of) gene clusters were also present in *C. jejuni* non-HS:19. For example, the sialyltransferase gene *cst-I* of cluster VI (Table 2, indicated in yellow), was detected in 5 % of the non-HS:19 genomes, particularly, as single gene, in genomes with ST-607 and ST-403 but also randomly distributed. It is unclear if the presence of a single gene, that is a part of a cluster in *C. jejuni* HS:19, will be functional or may contribute to GBS in non-HS:19 strains. The complete cluster VI was only sporadically found in non-HS:19 genomes, but in 8 % of these genomes, cluster VI was partly (four genes, *CjjRM3420_1435-1438*, Table 2) present.

Additionally, besides present in genomes of *C. jejuni* HS:19, the complete cluster X was also positively and almost exclusively related to *C. jejuni* with the ST-677 genetic background.

Due to the inability to produce sialylated LOS (ST-677 stains have LOS class E), ST-677 is not related to GBS. Cluster X may contribute to virulence as ST-677 stains are associated with bacteraemia and prolonged hospitalization [43, 44]. The genes *CjjRM3420_1440, CjjRM3420_1447* and *CjjRM3420_1449,* were identified only sporadically (<0.4 %) and randomly in the non-HS:19 genomes (Table 2). Besides HS:19 capsular genes, these genes may be used as markers for *C. jejuni* HS:19.

### HS:19 Methylomes

Because several methyltransferases were amongst the genes that were identified as significantly associated with *C. jejuni* HS:19, a methylation pattern-analysis was performed on PacBio sequence data that were available for two strains (RM1245 and RM1477). The methylomes of these strains exhibited a common *Campylobacte* r methylation of the 5′-RA^m6^ ATTY-3′ motif (Table 3) [16, 45]. The methylation of the 5′-RA^m6^ ATTY-3′ motif is likely related to the presence of the *Cj0208* methyltransferase gene homologues within these two strains (homologue to *CjjRM3420_0203*). Both HS:19 strains also exhibited the methylation of the 5′-C^m6^ ATG-3′ motif. The 5′-C^m6^ ATG-3′ methylation is consistent with a D12-class N6-adenine-specific DNA methyltransferase. A REBASE query with the sequences of the HS:19-related methyltransferases revealed that gene *CjjRM3420_1718* of cluster X encodes for this specificity. This indicates that, besides *C. jejuni* HS:19, *C. jejuni* ST-677, which contains a homologue of gene *CjjRM3420_1718* (cluster X, Fig. 3, Table 3), may also have this 5′-C^m6^ ATG-3′ methylation. The 5′-C^m6^ ATG-3′ methylation was not observed in four *C. jejuni* non-HS:19/non-ST-677 strains, including strains NCTC 11168 and 81–176 [45]. Strains RM1245 and RM1477 also exhibit a novel 5′-^m6^ AGTNNNNNNRTTG-3′ methylation motif that, unfortunately, could not be appointed to a particular methyltransferase gene by REBASE. The three methylation motifs were randomly distributed over the genomes. Further research is needed to determine if *C. jejuni* HS:19 specific methylation motifs contribute to virulence and GBS.

**Table 3.** Methylation profiles of *C. jejuni* strains RM1245 and RM1477. Methylated bases (m6A) are indicated in bold

Strain	*CjjRM1245*			*CjjRM1477*		
	Total motifs	Total methylated motifs	% of methylated motifs	Total motifs	Total methylated motifs	% of methylated motifs
5′-RA**^m6^A**TTY-3′	27.594	26.952	97.7	28 000	27.623	98.7
5′-C**^m6^AT** G-3′	6.266	6.207	99.1	6.404	6.377	99.6
5′-**^m6^A**GTNNNNNNRTTG-3′	316	310	98.1	325	325	100

## Discussion

In the present study, we demonstrate that *C. jejuni* HS:19 strains have unique genes and gene clusters that encode proteins involved in persistence, restriction-modification, methylation and biomolecule sulphation. A cluster of linked genes that encoded proteins involved in biomolecule sulphation also contained a gene encoding the campylobacter sialyltransferase Cst-I. The location of this gene cluster, just downstream of the capsule biosynthesis locus, suggests that sialic acid and sulphate could be modifications of the polysaccharide capsule.

In full agreement with previous reports we show that *C. jejuni* serotype HS:19 strains are genetically conserved [46], have a LOS locus class A [1, 47], and belong to MLST clonal complex ST-22 [7, 48, 49] (PubMLST database).

Our observation that *C. jejuni* HS:19 strains have extra clusters of genes that encode restriction-modification systems (protecting bacteria from incoming foreign DNA) may explain why the gene content of these strains is so conserved. The presence of CJIE1, containing a nuclease (DNS) that inhibits natural transformation [50], is another potential reason for genome preservation, although only 4/15 *C. jejuni* HS:19 strains possessed the integrated element.

We concluded that, among the HS:19 serotype, GBS-related strains could not be discriminated from enteritis-related strains. Specifically, we did not find genomic rearrangements, presence/absence of CJIEs, SNPs within the LOS or capsule locus, or variability in poly-G/C tracts of phase-variable genes that were associated consistently with either clinical outcome. The number of strains we tested was relatively low (n=15). To see if our findings can be confirmed, it would be worthwhile to perform a similar study with a larger number of *C. jejuni* HS:19 strains derived from GBS and enteritis patients.

With no apparent difference between GBS and enteritis (only)-related *C. jejuni* strains, the question remained: could the identified genes or gene clusters contribute to the increased risk of developing GBS, for example, by being involved in promoting infection, activation or modulation of the immune response, or some other reason?

Besides preventing the integration of foreign DNA, restriction-modification systems can also regulate virulence. Methylation of adenine, for example, can regulate the expression of genes involved in motility, host colonization, invasion of intestinal epithelial cells and M-cell cytotoxicity [51–53]. Enhanced colonization and invasiveness of *C. jejuni* can result in systemic invasive disease with post-infectious complications as a consequence. We determined that *CjjRM3420_1718* of cluster X encodes a methyltransferase able to methylate the adenine in the sequence 5′-CATG-3′. Additionally, a novel 5′-^m6^ AGTNNNNNNRTTG-3 methylation motif was identified. Our analysis provides the first methylated sequence motifs within *C. jejuni* HS:19 strains. It is possible that the methylation patterns only apply to the two strains that were analysed. However, *C. jejuni* HS:19 strains are highly conserved and the gene analogue to CjjRM3420_1718 is present in all *C. jejuni* HS:19 strains indicating that the observed methylation patterns are probably a feature of all HS:19 strains. Although of interest, the potential biological roles of these motifs, and additional research that may demonstrate a connection to GBS is beyond the scope of this study.

One of the genes significantly associated with *C. jejuni* HS:19 was *cst-I. In vivo*, it is not known what the target of this transferase is, but *in vitro*, it was established that the purified Cst-I protein could add sialic acid to a galactose acceptor [54]. With this *in vitro* functionality, Cst-I behaves similarly to Cst-II, known to transfer sialic acid to galactose on the outer core LOS. The cholera toxin and sialoadhesin binding experiments with a *cst-II* knockout mutant, however, showed that Cst-I does not add sialic acid to the LOS, resulting in the production of ganglioside-mimicking structures such as GM1a and GD1a. This implies that Cst-I may sialylate an acceptor outside of the LOS.

In the genome, *cst-I* is only seven genes removed from the upstream capsule biosynthesis locus, and downstream close to two glycosyltransferase genes (*CjjRM3420_1440* and *1451*) and two capsule biosynthesis encoding genes (*CjjRM3420_1452* and *1453*). The proximity of *cst-I* to the capsular biosynthesis locus and additional capsular genes suggest that Cst-I may sialylate the polysaccharide capsule. But previous mass-spectrometry analysis of a *C. jejuni* HS:19 strain did not show that sialic acid was a part of the molecular capsular structure [55]. It is possible that the sialic acid residues were lost during mass-spectrometry sample preparation because it is known that sialic acid residues are unstable and easily dissociate from the capsular polysaccharide [56]. More recently, a sialic acid stabilization technique (permethylation) [57] was described. It is worthwhile to re-assess the presence of sialic acid modifications in the *C. jejuni* HS:19 capsule using this technique. The *cst-I* gene was also found in 5 % of non-HS:19 genomes, examined in this study, including in genomes with ST-403, serotype HS:23 and LOS locus class B. C *. jejuni* with these features have been associated with GBS in Bangladesh [49].

We annotated gene *CjjRM3420_1441* of cluster VI as an anaerobic sulphate-maturating enzyme. The function of this gene in *C. jejuni* is unclear. Still, for the intestinal commensal *Bacteroides thetaiotaomicron,* it was shown that an orthologue of this enzyme activates sulphatases through a post-translational modification [42]. The activity appears to be related to the presence of sulphated mucins [42], which are present in the intestine and are a nutrient source for C *ampylobacter* [58]. Based on the annotated functions of the genes in cluster VI, which also codes for sulphate transport and sulphate modification, we propose that in the intestine, *C. jejuni* HS:19 strains utilize the sulphate, that becomes available after sulphatase activity for the modification of biomolecules.

The presence of a putative, sulphotransferase (*CjjRM3420-1439*) implies that *C. jejuni* can incorporate the sulphate groups into its proteins or glycan structures, including the capsule and the LOS. In combination with a sialyltransferase (Cst-I or Cst-II), novel sulphated ganglioside-mimicking structures may be formed that could cause GBS. The observation that antibodies directed to sulphated glycan structures, including sulphated gangliosides, are abundantly present in serum derived from patients with GBS [59] strengthens the concept that such structures may exist on pathogens that cause GBS. Specific recognition of sulphated biomolecules may also modulate the immune response in the direction of GBS as several immunologically relevant receptors, including l-selectin and certain sialic acid-binding immunoglobulin-like lectins (siglecs) have an affinity for sulphated ligands [60, 61].

Besides the cluster of genes containing *cst-I* and the sulphation pathway genes, another gene cluster (cluster VII, CjjRM3420_1447-1454), close to the capsule biosynthesis locus, is also involved in capsule biosynthesis and modification. Bacterial capsules are generally accepted as an essential virulence factor that protects bacteria from complement-mediated killing, the first line of defence against invading pathogens [62]. Bacteria, including *C. jejuni,* that lack capsule are significantly more sensitive to complement-mediated killing than encapsulated bacteria [63, 64]. Changes in capsular carbohydrate branching and modifications within the capsular structure can also affect other immune responses [65]. It, for example, was shown that an O-methyl phosphoramidate (MeOPN) modification found on ~75 % of *C. jejuni* capsules, including capsules of *C. jejuni* HS:19, modulates cytokine release by murine dendritic cells [66]. Moreover, *in vivo*, infection with a MeOPN mutant strain leads to higher levels of IL-17 compared to the wild-type strain [67, 68]. IL-17 signalling contributes to autoimmune diseases and is indicated to play a role in GBS [69, 70]. In summary, we propose genes in cluster VII as candidates that may also contribute to capsule modifications leading to complement resistance or immune modulation, which may shift immune responses towards GBS.

The identified cluster III of *C. jejuni* HS:19, involved in flagellar biosynthesis, showed genes with >20 % gene sequence variability compared to similar genes in *C. jejuni* non-HS:19. It is unclear how such variability may contribute to virulence leading to GBS. In contrast to the flagella of several other bacterial species, *C. jejuni* flagella, including flagella of *C. jejuni* HS:19 (strain GB18) do not activate the immune system through TLR5 [71]. Despite this, *C. jejuni* flagella are a dominant immunogen in humans, as demonstrated by seroconversion in patients after infection [72, 73]. Of course, further future experimental validation is pivotal to determine if there is a causal relation between the *C. jejuni* HS:19-related genes and gene cluster identified, and GBS.

In conclusion, we identified unique genes and gene clusters and novel methylation patterns in *C. jejuni* serotype HS:19 strains. This study provides the first systematic evaluation of *C. jejuni* serotype HS:19 genomics compared to an extensive collection of non*-*HS:19 strains. The identification of novel genes associated with sulphate acquisition and utilization in HS:19 strains demonstrate a possible contribution of strain-specific factors in the development of the sequelae. Our findings provide novel insight into virulence factors of *C. jejuni* serotype HS:19 that may explain why infections with such strains impose an increased risk for GBS.

## Supplementary Data

Supplementary material 1
