# Supplementary material for: Biomolecule sulphation and novel methylations related to Guillain-Barré syndrome-associated Campylobacter jejuni serotype HS:19
Source: Microb Genom. 2021 Nov 1;7(11):000660. doi: 10.1099/mgen.0.000660 (PMC8743553; doi:10.1099/mgen.0.000660)
Supplement: Supplementary material 1 [file mgen-7-0660-s001.pdf]

Supplemental Tables, Figures and Data.

Supplemental Table 1. Repository information for the genomes sequenced in this study.

SUBID	BioProject	BioSample	Localid	Accession	Organism
SUB9233512	PRJNA634604	SAMN15004773	CjGB03	CP071579	*Campylobacter jejuni* GB03
SUB9228550	PRJNA634604	SAMN15004774	CjGB18	CP071577	*Campylobacter jejuni* GB18
SUB9233606	PRJNA634604	SAMN15004775	CjGB28	CP071582	*Campylobacter jejuni* GB28
SUB9233607	PRJNA634604	SAMN15004776	CjGB60	CP071583	*Campylobacter jejuni* GB60
SUB9220467	PRJNA634604	SAMN15004779	CjRM1510	CP071574	*Campylobacter jejuni* RM1510
SUB9228496	PRJNA634604	SAMN15004780	CjRM3147	CP071575	*Campylobacter jejuni* RM3147
SUB9228544	PRJNA634604	SAMN15004781	CjEnt12	CP071576	*Campylobacter jejuni* R12
SUB9233595	PRJNA634604	SAMN15004782	CjEnt23	CP071580	*Campylobacter jejuni* R23
SUB9233601	PRJNA634604	SAMN15004783	CjEnt31	CP071581	*Campylobacter jejuni* R31
SUB9233425	PRJNA634604	SAMN15004784	CjEnt72	CP071578	*Campylobacter jejuni* R72
SUB9233611	PRJNA634604	SAMN15004785	CjEnt73	CP071584	*Campylobacter jejuni* R73
SUB9235759	PRJNA348673	SAMN05871073	CjGB19	CP071592	*Campylobacter jejuni subsp. jejuni* GB19
SUB9235759	PRJNA348673	SAMN05871073	CjGB19pGB19	CP071593	Campylobacter jejuni *subsp. jejuni* GB19
SUB9235875	PRJNA348675	SAMN05871074	CjGB26	CP071585	*Campylobacter jejuni subsp. jejuni* GB26

Supplemental Table 2. The presence of *Campylobacter jejuni* integrated elements (CJIE)s in strains with the capsular Penner serotype HS:19. Mauve alignment and BLASTn analysis was used to identify and characterise CJIEs in *C. jejuni*.

Strain	Disease	CJIE present	Homology with	Location in genome (bp)	Size (bp)	Integration site	Naturally transformable
GB03	GBS	yes	CJIE1	185,572-225,032	39.461	in a putative protease	no
GB18	GBS	no	-	-	-	-	yes
GB28	GBS	no	-	-	-	-	yes
GB60	GBS	no	-	-	-	-	yes
RM1245	GBS	no	-	-	-	-	no
RM1477	GBS	yes	CJIE1	1,500,862-1,538,087	37.226	after ABC transporter	no
RM1510	GBS	yes	CJIE1	821,493-858,403	36.911	after putative sulphortransferase	no
RM3147	GBS	no	-	-	-	-	yes
R12	Enteritis	yes	CJIE2	452,580-492,176	39.597	in tRNA Arg-CCT	yes
R23	Enteritis	no	-	-	-	-	yes
R31	Enteritis	no	-	-	-	-	yes
R72	Enteritis	no	-	-	-	-	yes
R73	Enteritis	no	-	-	-	-	yes
RM3420	Enteritis	yes	CJIE1	1,326,743-1,365,298	38.556	in putative periplasmic protein	no
RM1285	chicken isolate	yes	unique	879,831-919,449	39.619	in tRNA Leu-GAG	yes

Supplemental Table 3. Genes in *C. jejuni* strain *CjjRM3420* that contain poly G/C-tracts.

Gene, gene product function, location of poly G/C-tract within the gene, status of the poly G/C-tract and numbers of poly G/C-tracts that are in an in-frame status (GBS vs non-GBS).

Grey rows indicate poly G/C-tract information for genes within a gene cluster involved in a similar function.

[a], non-annotated pseudogene between CjjRM3420_0171 and _0171 containing a poly G/C-tract;

[b], these genes are sometimes identical but can have different sizes and annotations, the largest annotated CDS is 2604;

[c],poly G-tract is close to the 3' end the CDS and produces products of varying size when variable; IF, CDS in frame; OOF, CDS out of frame; OOFB, CDS out of frame before poly G/C-tract; IF/OOF, variable poly G-tract with in frame and out frame CDS states; IF/no polyG, CDS in frame, no poly G/C-tracts; GGGGAGGGG, poly G/C-tract altered to sequence given; GGGCGGGGGG, poly G/C-tract altered to sequence given, 9bp, 10bp, 9/10bp, 10/11bp, 12/13bp, sizes of intragenic poly G/C- tract upstream off CjjRM3420_1407, NP, not present; AP, not applicable.

Gene	Gene product function	CjjRM3420		CDS status															(partial) IF GBS	(partial) IF Enteritis/Food	*p* (Fisher exact)
				GBS-related (n = 8)								Enteritis/food-related (n = 7)									
		gene length (bp)	start G/C-tract	GB03	GB18	GB28	GB60	RM1245	RM1477	RM1510	RM3147	R12	R23	R31	R72	R73	RM1285	RM3420			
CjjRM3420_0039	DNA cleavage	3738	2563	IF/no polyG	IF/no polyG	IF	IF/no polyG	IF/OOF	IF/OOF	IF/OOF	IF/no polyG	OOFB	IF/no polyG	IF/no polyG	IF/GGGGAGGGG	IF/no polyG	IF/no polyG	IF/OOF	8	6	0.4667
CjjRM3420_0053	hypothetical protein	720[c]	708	IF	OOFB	IF	OOFB	IF	IF	IF	IF	IF	OOFB	OOFB	IF	OOFB	IF	IF	6	4	0.6084
CjjRM3420_0171-0172 [a]	pseudogene	NA	NA	NA	NA	NA	NA	NA	NA	NA	NA	NA	NA	NA	NA	NA	NA	NA	NA	NA	NA
CjjRM3420_0472 [b]	unknow n	1170	30	IF/OOF	IF/OOF	OOF	IF	IF/OOF	IF/OOF	IF/OOF	OOF/GGGGAGGGG	OOF	OOF/GGGGAGGGG	IF/OOF	OOF/GGGGAGGGG	IF	OOF/GGGGAGGGG	OOF	6	2	0.1319
CjjRM3420_0590	DUF2920 domain protein	1263	584	OOF	IF	IF	IF	IF/OOF	IF/OOF	IF/OOF	IF	OOF	IF	IF	IF	IF	IF/OOF	IF	7	6	1
CjjRM3420_0649	capsule modification	1395	835	IF/OOF	IF	IF	IF/OOF	IF/OOF	IF/OOF	IF/OOF	IF	IF	IF	IF/OOF	IF/OOF	IF/OOF	IF	IF	8	7	1
CjjRM3420_0931 [b]	unknow n	1170	30	IF/OOF	IF/OOF	IF/OOF	IF/OOF	IF/OOF	IF/OOF	IF	OOF/GGGGAGGGG	OOF	OOF/GGGGAGGGG	IF/OOF	OOF/GGGGAGGGG	IF/OOF	OOF/GGGGAGGGG	IF	7	4	0.2821
CjjRM3420_1249	flagella biosynthesis	1308	143	IF	IF/OOF	IF/OOF	IF/OOF	IF/OOF	IF/OOF	IF	IF	IF	IF/OOF	IF/OOF	IF/OOF	IF/OOF	IF	IF	8	7	1
CjjRM3420_1250		794	309	OOF	IF/OOF	IF/OOF	IF/OOF	IF/OOF	IF/OOF	OOF	IF	OOF	IF/OOF	OOF	OOF	IF/OOF	OOF	OOF	6	2	0.1319
CjjRM3420_1258		1215	587	IF/OOF	IF/OOF	IF/OOF	IF/OOF	IF	IF	OOF	OOF	IF	IF/OOF	IF/OOF	IF	IF/OOF	IF	IF	6	7	0.4667
CjjRM3420_1260		1209	576	NP	IF/OOF	IF/OOF	NP	IF	IF	IF	IF	NP	NP	NP	IF	NP	IF/OOF	OOF	6	2	0.1319
CjjRM3420_1264		1215	578	IF	IF	OOF	IF/OOF	IF	IF	OOF	IF	IF	OOF	IF	IF	IF	IF	OOF	6	5	1
CjjRM3420_1278		1245	560	IF	IF/OOF	IF/OOF	IF/OOF	IF	IF/OOF	OOF	IF	IF	IF	IF	IF	IF/OOF	IF	IF	7	7	1
CjjRM3420_1407	capsule biosynthesis	1407	-21	10/11bp	10/11bp	9/10bp	10/11bp	12/13bp	10/11bp	10bp	9bp	10bp	10/11bp	10bp	10/11bp	10/11bp	10/11bp	9bp	NA	NA	NA
CjjRM3420_1414	capsule modification (MeOPn)	774	393	IF	IF	OOF	IF	IF	IF/OOF	IF	OOF	IF/OOF	IF	IF	IF	IF	IF	IF	6	7	0.4667
CjjRM3420_1415		1833	93	IF	IF/OOF	IF/OOF	IF	IF/OOF	IF/OOF	OOF	OOF	IF/OOF	IF/OOF	IF	IF/OOF	IF/OOF	IF/OOF	IF/OOF	6	7	0.4667
CjjRM3420_1434	sialylation	1278	1206	IF/OOF	IF	IF/OOF	IF/OOF	IF/no polyG	IF/GGGGAGGGG	IF/GGGGAGGGG	IF	IF/GGGGAGGGG	IF/OOF	IF/OOF	IF	IF	IF/OOF	IF/GGGGAGGGG	8	7	1
CjjRM3420_1439	sulfation	1203[c]	1146	IF	IF	IF	IF	IF	IF	IF	IF	IF	IF	IF	IF	IF	IF	IF	8	7	
CjjRM3420_1441		960	138	IF	IF	IF	IF/OOF	IF/OOF	IF	IF	IF	IF/OOF	OOF	IF/OOF	IF	IF/OOF	IF/OOF	IF	8	6	0.4667
CjjRM3420_1448	methylation	1137	539	IF	IF/OOF	IF	IF	IF	IF/OOF	IF	IF	IF/OOF	IF	IF/OOF	IF/OOF	IF/OOF	OOF	IF/OOF	8	6	0.4667
CjjRM3420_1454	methylation	1203	126	IF/OOF	IF/OOF	IF/OOF	IF/OOF	IF/OOF	IF/OOF	IF/OOF	IF/OOF	IF/OOF	IF/OOF	IF/OOF	IF/OOF	OOF	IF/OOF	OOF	8	5	0.2
CjjRM3420_1567	putative 4Fe-4S dicluster domain dimethylsulfoxide (pseudogene)	673	153	IF	IF/OOF	IF/OOF	IF/OOF	OOF	OOF	OOF	OOF	OOF/GGGCGGGGGG	IF	IF/OOF	OOF	OOF	OOF	OOF	4	2	0.6084

Supplemental Table 4. Blast analysis of ST-22 strains derived from PubMLST. To determine whether ST-22 genomes generally have capsular Penner serotype HS:19, LOS class A1 and the clusters identified in this study, ST-22 genomes with a core genome MLST (cgMLST) that had less than 200 cgMLST allelic differences were selected in PubMLST and blasted (BLASTn) against sequences relevant for this study (Supplemental data 1). Green, indicates that the queried sequence was present; yellow, indicates that the queried sequence was absent (based on >98% identity and >60% query coverage).

Isolate id	Isolate	Country	Year	Disease	MLST-ST	MLST-CC	HS:19	LOS class A1	Cluster I	Cluster II	Cluster III	Cluster IV	Cluster VI	Cluster X
1818	D5476	USA	1998	enteritis	22	ST-22	1	1	1	1	1	1	1	1
1820	D0445	USA	1983	enteritis	22	ST-22	1	1	1	1	1	1	1	1
6199	OXC342	UK [England]	2004	enteritis	22	ST-22	1	1	1	1	1	1	1	1
14651	OXC2413	UK [England]	2007	enteritis	22	ST-22	1	1	1	1	1	1	1	1
16061	OXC6264	UK [England]	2011	enteritis	22	ST-22	1	1	1	1	1	1	1	1
16390	OXC6641	UK [England]	2011	enteritis	22	ST-22	1	1	1	1	1	1	1	1
18201	OXC6644	UK [England]	2011	enteritis	22	ST-22	1	1	1	1	1	1	1	1
18202	OXC6645	UK [England]	2011	enteritis	22	ST-22	1	1	1	1	1	1	1	1
21585	OXC6866	UK [England]	2012	enteritis	22	ST-22	1	1	1	1	1	1	1	1
22083	OXC6887	UK [England]	2012	enteritis	22	ST-22	1	1	1	1	1	1	1	1
22084	OXC6888	UK [England]	2012	enteritis	22	ST-22	1	1	1	1	1	1	1	1
22097	OXC6902	UK [England]	2012	enteritis	22	ST-22	1	1	1	1	1	1	1	1
22269	OXC7085	UK [England]	2012	enteritis	22	ST-22	1	1	1	1	1	1	1	1
22669	OXC7178	UK [England]	2012	enteritis	22	ST-22	1	1	1	1	1	1	1	1
23903	OXC7364	UK [England]	2012	enteritis	22	ST-22	1	1	1	1	1	1	1	1
23906	OXC7367	UK [England]	2012	enteritis	22	ST-22	1	1	1	1	1	1	1	1
23913	OXC7375	UK [England]	2012	enteritis	22	ST-22	1	1	1	1	1	1	1	1
23992	OXC7463	UK [England]	2012	enteritis	22	ST-22	1	1	1	1	1	1	1	1
24018	OXC7490	UK [England]	2012	enteritis	22	ST-22	1	1	1	1	1	1	1	1
24607	OXC7809	UK [England]	2013	enteritis	22	ST-22	1	1	1	1	1	1	1	1
24635	OXC7838	UK [England]	2013	enteritis	22	ST-22	1	1	1	1	1	1	1	1
24945	OXC7697	UK [England]	2013	enteritis	22	ST-22	1	1	1	1	1	1	1	1
25568	OXC7942	UK [England]	2013	enteritis	22	ST-22	1	1	1	1	1	1	1	1
25593	OXC8043	UK [England]	2013	enteritis	22	ST-22	1	1	1	1	1	1	1	1
25980	Dg253	UK			22	ST-22	1	1	1	1	1	1	1	1
26044	Dg258	UK			22	ST-22	1	1	1	1	1	1	1	1
30554	OXC8868	UK [England]	2014	enteritis	22	ST-22	1	1	1	1	1	1	1	1
30815	OXC9136	UK [England]	2014	enteritis	22	ST-22	1	1	1	1	1	1	1	1

30821	OXC9142	UK [England]	2014	enteritis	22	ST-22	1	1	1	1	1	1	1	1
30918	OXC9245	UK [England]	2014	enteritis	22	ST-22	1	1	1	1	1	1	1	1
31162	OXC9343	UK [England]	2014	enteritis	22	ST-22	1	1	1	1	1	1	1	1
31420	OXC9601	UK [England]	2015	enteritis	22	ST-22	1	1	1	1	1	1	1	1
31500	OXC9681	UK [England]	2015	enteritis	22	ST-22	1	1	1	1	1	1	1	1
31556	H144520622	UK [England]	2014	enteritis	22	ST-22	1	1	1	1	1	1	1	1
32554	H150260421	UK [England]	2015	enteritis	22	ST-22	1	1	1	1	1	1	1	1
32824	OXC9768	UK [England]	2015	enteritis	22	ST-22	1	1	1	1	1	1	1	1
34427	OXC9895	UK [England]	2015	enteritis	22	ST-22	1	1	1	1	1	1	1	1
34441	OXC9909	UK [England]	2015	enteritis	22	ST-22	1	1	1	1	1	1	1	1
35305	Cj1997-11	USA			22	ST-22	1	1	1	1	0	1	1	1
38448	ARI1517	UK [Scotland]	2012		22	ST-22	1	1	1	1	1	1	1	1
38473	ARI1544	UK [Scotland]	2012		22	ST-22	1	1	1	1	1	1	1	1
38514	ARI1591	UK [Scotland]	2012		22	ST-22	1	1	1	1	1	1	1	1
38680	ARI1786	UK [Scotland]	2012		22	ST-22	1	1	1	1	1	1	1	1
38682	ARI1788	UK [Scotland]	2012		22	ST-22	1	1	1	1	1	1	1	1
38728	ARI1836	UK [Scotland]	2012		22	ST-22	1	1	1	1	1	1	1	1
39288	ARI2483	UK [Scotland]	2013		22	ST-22	1	1	1	1	1	1	1	1
40905	ARI3321	UK [Scotland]	2014	enteritis	22	ST-22	1	1	1	1	1	1	1	1
41650	C1018	UK [Scotland]	2014		22	ST-22	1	1	1	1	1	1	1	1
42172	ARI3891	UK [Scotland]	2015	enteritis	22	ST-22	1	1	1	1	1	1	1	1
43814	NWC161	UK [England]	2016	enteritis	8118	ST-22	1	1	1	1	1	1	1	1
47373	41974	USA	2011		22	ST-22	1	1	1	1	1	1	1	1
47709	NWC256	UK [England]	2016	enteritis	22	ST-22	1	1	1	1	1	1	1	1
47767	NWC280	UK [England]	2015	enteritis	22	ST-22	1	1	1	1	1	1	1	1
47791	OXC10181	UK [England]	2015	enteritis	22	ST-22	1	1	1	1	1	1	1	1
47794	NWC266	UK [England]	2015	enteritis	22	ST-22	1	1	1	1	1	1	1	1
48259	ARI3902	UK [Scotland]			22	ST-22	1	1	1	1	1	1	1	1
48304	ARI4172	UK [Scotland]	2015		22	ST-22	1	1	1	1	1	1	1	1
50282	NWC529	UK [England]	2015	enteritis	22	ST-22	1	1	1	1	1	1	1	1
50854	ACP163	UK	2016		22	ST-22	1	1	1	1	1	1	1	1
50932	ACP1278	UK	2016		22	ST-22	1	1	1	1	1	1	1	1
57331	OXC10470	UK [England]	2016	enteritis	22	ST-22	1	1	1	1	1	1	1	1
58092	NWC1010	UK [England]	2017	enteritis	22	ST-22	1	1	1	1	1	1	1	1
58157	ARI3973	UK [Scotland]	2015		22	ST-22	1	1	1	1	1	1	1	1
59035	OXC10639	UK [England]	2017	enteritis	22	ST-22	1	1	1	1	1	1	1	1
59520	OXC10732	UK [England]	2017	enteritis	22	ST-22	1	1	1	1	1	1	1	1
59522	NWC1327	UK [England]	2017	enteritis	22	ST-22	1	1	1	1	1	1	1	1
59617	NWC1280	UK [England]	2017	enteritis	22	ST-22	1	1	1	1	1	1	1	1
61015	M00214	USA	2007		22	ST-22	1	1	1	1	1	1	1	1
61177	OXC10848	UK [England]	2017	enteritis	22	ST-22	1	1	1	1	1	1	1	1

62693	NWC1741	UK [England]	2017	enteritis	22	ST-22	1	1	1	1	1	1	1	1
62696	NWC1738	UK [England]	2017	enteritis	22	ST-22	1	1	1	1	1	1	1	1
62729	NWC1740	UK [England]	2017	enteritis	22	ST-22	1	1	1	1	1	1	1	1
62917	NWC1877	UK [England]	2017	enteritis	8118	ST-22	1	1	1	1	1	1	1	1
62933	OXC11201	UK [England]	2017	enteritis	22	ST-22	1	1	1	1	1	1	1	1
69389	NWC1993	UK [England]	2017	enteritis	22	ST-22	1	1	1	1	1	1	1	1
69398	OXC11267	UK [England]	2017	enteritis	22	ST-22	1	1	1	1	1	1	1	1
69415	OXC11266	UK [England]	2017	enteritis	22	ST-22	1	1	1	1	1	1	1	1
69452	NWC1958	UK [England]	2017	enteritis	22	ST-22	1	1	1	1	1	1	1	1
69502	NWC1925	UK [England]	2017	enteritis	22	ST-22	1	1	1	1	1	1	1	1
70407	2010D-7920	USA		enteritis	22	ST-22	1	1	1	1	1	1	1	1
70410	2010D-7921	USA		enteritis	22	ST-22	1	1	1	1	1	1	1	1
70412	2005782	USA		enteritis	22	ST-22	1	1	1	1	1	1	1	1
70413	20070225	USA		enteritis	22	ST-22	1	1	1	1	1	1	1	1
70414	20070697	USA		enteritis	22	ST-22	1	1	1	1	1	1	1	1
70415	PNUSAC000105	USA	2015	enteritis	22	ST-22	1	1	1	1	0	1	1	1
70416	PNUSAC000107	USA	2015	enteritis	22	ST-22	1	1	1	1	1	1	1	1
70421	PNUSAC000310	USA	2016	enteritis	22	ST-22	1	1	1	1	1	1	1	1
70809	NWC2343	UK [England]	2018	enteritis	8118	ST-22	1	1	1	1	1	1	1	1
76108	CjRM1477	USA		GBS	22	ST-22	1	1	1	1	1	1	1	1
76110	CjRM1510	Japan		GBS	22	ST-22	1	1	1	1	1	1	1	1
76878	OXC11712	UK [England]	2018		22	ST-22	1	1	1	1	1	1	1	1
76960	NWC2662	UK [England]	2018	enteritis	22	ST-22	1	1	1	1	1	1	1	1
76976	NWC2661	UK [England]	2018	enteritis	22	ST-22	1	1	1	1	1	1	1	1
77030	NWC2694	UK [England]	2018	enteritis	22	ST-22	1	1	1	1	1	1	1	1
77069	NWC2715	UK [England]	2018	enteritis	22	ST-22	1	1	1	1	1	1	1	1
77157	OXC11837	UK [England]	2018		22	ST-22	1	1	1	1	1	1	1	1
77254	OXC11872	UK [England]	2018		22	ST-22	1	1	1	1	1	1	1	1
78185	FDI670	UK	2018		22	ST-22	1	1	1	1	1	1	1	1
78273	OXC11922	UK [England]	2018		22	ST-22	1	1	1	1	1	1	1	1
78465	NWC2908	UK [England]	2018	enteritis	22	ST-22	1	1	1	1	1	1	1	1
79189	CVM N17C205	USA	2017		22	ST-22	1	1	1	1	1	1	1	1
79244	PS00268	USA	2017		22	ST-22	1	1	1	1	1	1	1	1
80426	npCAMPY38	Peru	2013	carrier	22	ST-22	1	1	1	1	1	1	1	1
80470	p3H2	Peru	2012	carrier	22	ST-22	1	1	1	1	1	1	1	0
80828	CVM N18C436	USA	2018		22	ST-22	1	1	1	1	1	1	1	1
80871	PNUSAC009624	USA			22	ST-22	1	1	1	1	1	1	1	0
80937	PNUSAC009814	USA			22	ST-22	1	1	1	1	1	1	1	1
80957	PNUSAC009671	USA			22	ST-22	1	1	1	1	0	1	1	0
80958	PNUSAC009677	USA			22	ST-22	1	1	1	1	1	1	1	1
81073	PNUSAC009524	USA			22	ST-22	1	1	1	1	1	1	1	1

81074	PNUSAC009523	USA	22	ST-22	1	1	1	1	1	1	1	1
81101	PNUSAC009435	USA	22	ST-22	1	1	1	1	1	1	1	0
81126	PNUSAC009493	USA	22	ST-22	1	1	1	1	1	1	1	1
81212	PNUSAC005995	USA	22	ST-22	1	1	1	1	1	1	1	0
81409	PNUSAC005599	USA	22	ST-22	1	1	1	1	1	1	1	1
81423	PNUSAC005609	USA	22	ST-22	1	1	1	1	1	1	1	1
81435	PNUSAC005719	USA	22	ST-22	1	1	1	1	1	1	1	1
81497	PNUSAC005694	USA	22	ST-22	1	1	1	1	1	1	1	1
81518	PNUSAC005640	USA	22	ST-22	1	1	0	1	1	1	1	1
81526	PNUSAC005591	USA	22	ST-22	1	1	1	1	1	1	1	1
81695	PNUSAC005521	USA	22	ST-22	1	1	1	1	1	1	1	0
81780	PNUSAC005448	USA	22	ST-22	1	1	1	1	1	1	1	1
81802	PNUSAC005440	USA	22	ST-22	1	1	1	1	1	1	1	1
81863	PNUSAC005182	USA	22	ST-22	1	1	1	1	1	1	1	1
81869	PNUSAC005183	USA	22	ST-22	1	1	1	1	1	1	1	1
81890	PNUSAC005191	USA	22	ST-22	1	1	1	1	1	1	1	1
81916	PNUSAC005233	USA	22	ST-22	1	1	1	1	0	1	1	1
81920	PNUSAC005315	USA	22	ST-22	1	1	1	1	1	1	1	1
81953	PNUSAC005235	USA	22	ST-22	1	1	1	1	1	1	1	1
81965	PNUSAC005050	USA	22	ST-22	1	1	1	1	1	1	1	1
81976	PNUSAC005055	USA	22	ST-22	1	1	1	1	1	1	1	1
82088	PNUSAC005048	USA	22	ST-22	1	1	1	1	1	1	1	1
82401	PNUSAC004863	USA	22	ST-22	1	1	1	1	1	1	1	1
82600	D0448	USA	22	ST-22	1	1	1	1	1	1	1	1
82602	D0450	USA	22	ST-22	1	1	1	1	1	1	1	1
82730	PNUSAC009187	USA	22	ST-22	1	1	1	1	1	1	1	1
82783	PNUSAC001787	USA	22	ST-22	1	1	1	1	1	1	1	1
82852	PNUSAC001786	USA	22	ST-22	1	1	1	1	1	1	1	1
82853	PNUSAC001785	USA	22	ST-22	1	1	1	1	1	1	1	1
82854	PNUSAC001788	USA	22	ST-22	1	1	1	1	1	1	1	1
82862	PNUSAC001761	USA	22	ST-22	1	1	1	1	1	1	1	1
82888	PNUSAC002261	USA	22	ST-22	1	1	1	1	1	1	1	1
82936	2016D-0264	USA	22	ST-22	1	1	1	1	1	1	1	1
82998	2016D-0009	USA	22	ST-22	1	1	1	1	1	1	1	1
83130	PNUSAC008977	USA	22	ST-22	1	1	1	1	1	1	1	1
83147	PNUSAC002042	Unknown	22	ST-22	1	1	1	1	1	1	1	1
83228	PNUSAC004231	USA	22	ST-22	1	1	1	1	1	1	1	1
83262	PNUSAC001519	USA	22	ST-22	1	1	1	1	1	1	1	1
83276	PNUSAC001864	USA	22	ST-22	1	1	1	1	1	1	1	1
83303	PNUSAC002448	USA	22	ST-22	1	1	1	1	1	1	1	1
83320	PNUSAC002733	USA	22	ST-22	1	1	1	1	0	1	1	1

83477	PNUSAC002347	USA	22	ST-22	1	1	1	1	1	1	1	1
83571	PNUSAC001395	USA	22	ST-22	1	1	1	1	1	1	1	1
83619	PNUSAC002897	USA	22	ST-22	1	1	1	1	1	1	1	1
83796	PNUSAC002099	USA	22	ST-22	1	1	1	1	1	1	1	1
83965	PNUSAC000701	USA	22	ST-22	1	1	1	1	1	1	1	1
84030	PNUSAC001392	USA	22	ST-22	1	1	1	1	1	1	1	1
84059	PNUSAC002018	USA	22	ST-22	1	1	1	1	1	1	1	1
84078	2016D-0263	USA	22	ST-22	1	1	1	1	1	1	1	1
84086	PNUSAC001243	USA	22	ST-22	1	1	1	1	1	1	1	1
84147	PNUSAC001191	USA	22	ST-22	1	1	1	1	1	1	1	1
84168	PNUSAC001423	USA	22	ST-22	1	1	1	1	1	1	1	1
84305	PNUSAC003105	USA	22	ST-22	1	1	1	1	1	1	1	1
84415	2015D-0237	USA	22	ST-22	1	1	1	1	1	1	1	1
84492	TESTWGCAMPY0003880	USA	22	ST-22	1	1	1	1	1	1	1	1
84624	TESTWGCAMPY0002422	USA	22	ST-22	1	1	1	1	1	1	1	1
84661	PNUSAC000106	USA	22	ST-22	1	1	1	1	1	1	1	1
84754	PNUSAC001410	USA	22	ST-22	1	1	1	1	1	1	1	1
84778	PNUSAC001443	USA	22	ST-22	1	1	1	1	1	1	1	1
84842	PNUSAC001491	USA	22	ST-22	1	1	1	1	1	1	1	1
84843	PNUSAC001490	USA	22	ST-22	1	1	1	1	1	1	1	1
84887	2016D-0265	USA	22	ST-22	1	1	1	1	1	1	1	1
84992	PNUSAC000932	USA	22	ST-22	1	1	1	1	1	1	1	1
85018	PNUSAC000817	USA	22	ST-22	1	1	1	1	1	1	1	1
85151	TESTWGCAMPY0003883	USA			1	1	1	1	1	1	1	1
85168	TESTWGCAMPY0003884	USA	22	ST-22	1	1	1	1	1	1	1	1
85210	PNUSAC000532	Unknown	22	ST-22	1	1	1	1	1	1	1	1
85285	PNUSAC000173	USA	22	ST-22	1	1	1	1	1	1	1	1
85299	TESTWGCAMPY0002132	USA	22	ST-22	1	1	1	1	1	1	1	1
85350	TESTWGCAMPY0002420	USA	22	ST-22	1	1	1	1	1	1	1	1
85363	TESTWGCAMPY0002241	USA	22	ST-22	1	1	1	1	1	1	1	1
85806	PNUSAC003145	USA	22	ST-22	1	1	1	1	1	1	1	1
85879	PNUSAC004581	USA	22	ST-22	1	1	1	1	1	1	1	1
86068	PNUSAC004222	USA	22	ST-22	1	1	1	1	1	1	1	1
86072	PNUSAC003566	USA	22	ST-22	1	1	1	1	1	1	1	1
86093	PNUSAC003626	USA	22	ST-22	1	1	1	1	1	1	1	1
86161	PNUSAC002723	USA	22	ST-22	1	1	1	1	1	1	1	1
86247	PNUSAC004140	USA	22	ST-22	1	1	1	1	1	1	1	1
86327	PNUSAC000854	USA	22	ST-22	1	1	1	1	1	1	1	1
86342	PNUSAC001134	USA	22	ST-22	1	1	1	1	1	1	1	1
86350	PNUSAC000773	USA	22	ST-22	1	1	1	1	1	1	1	0
86367	PNUSAC000548	USA	22	ST-22	1	1	1	1	1	1	1	1

86435	PNUSAC003023	USA	22	ST-22	1	1	1	1	1	1	1	1
86488	PNUSAC002447	USA	22	ST-22	1	1	1	1	1	1	1	1
86512	PNUSAC001859	USA	22	ST-22	1	1	1	1	1	1	1	1
86536	PNUSAC002974	USA	22	ST-22	1	1	1	1	1	1	1	1
86644	PNUSAC001525	USA	22	ST-22	1	1	1	1	1	1	1	1
86765	2017D-0010	USA	22	ST-22	1	1	1	1	1	1	1	1
86828	PNUSAC002590	USA			1	1	1	1	1	1	1	1
86881	PNUSAC003641	USA	22	ST-22	1	1	1	1	1	1	1	1
87072	PNUSAC003243	USA	22	ST-22	1	1	1	1	1	1	1	1
87194	PNUSAC002900	USA	22	ST-22	1	1	1	1	1	1	1	1
87212	PNUSAC002890	USA	22	ST-22	1	1	1	1	1	1	1	1
87408	PNUSAC002503	USA	22	ST-22	1	1	1	1	1	1	1	1
87419	PNUSAC002377	USA	22	ST-22	1	1	1	1	1	1	1	1
87422	PNUSAC002440	USA	22	ST-22	1	1	1	1	1	1	1	1
87477	PNUSAC002332	USA	22	ST-22	1	1	1	1	1	1	1	1
87479	PNUSAC002325	USA	22	ST-22	1	1	1	1	1	1	1	1
87548	PNUSAC002071	USA	22	ST-22	1	1	1	1	1	1	1	1
87642	PNUSAC001776	USA	22	ST-22	1	1	1	1	1	1	1	1
87668	PNUSAC001181	USA	22	ST-22	1	1	1	1	1	1	1	1
87725	2016D-0262	USA	22	ST-22	1	1	1	1	1	1	1	1
87760	PNUSAC000969	USA	22	ST-22	1	1	1	1	1	1	1	1
87765	PNUSAC000955	USA	22	ST-22	1	1	1	1	1	1	1	1
87793	PNUSAC000723	USA	22	ST-22	1	1	1	1	1	1	1	1
87909	PNUSAC000509	USA	22	ST-22	1	1	1	1	1	1	1	1
87964	TESTWGCAMPY0003882	USA	22	ST-22	1	1	1	1	1	1	1	1
88043	TESTWGCAMPY0002411	USA	22	ST-22	1	1	1	1	1	1	1	1
88071	TESTWGCAMPY0001882	USA	22	ST-22	1	1	1	1	1	1	1	1
88125	TESTWGCAMPY0001881	USA	22	ST-22	1	1	1	1	1	1	1	1
88239	PNUSAC008810	USA	22	ST-22	1	1	1	1	1	1	1	1
88259	PNUSAC008849	USA	22	ST-22	1	1	1	1	1	1	1	1
88278	PNUSAC008594	USA	22	ST-22	1	1	1	1	1	1	1	1
88741	PNUSAC007123	USA	22	ST-22	1	1	1	1	0	1	1	1
88990	PNUSAC008219	USA	22	ST-22	1	1	1	1	1	1	1	1
89146	PNUSAC008111	USA	22	ST-22	1	1	1	1	1	1	1	1
89238	PNUSAC007797	USA	22	ST-22	1	1	1	1	1	1	1	1
89259	PNUSAC008001	USA	22	ST-22	1	1	1	1	1	1	1	1
89376	PNUSAC007335	USA	22	ST-22	1	1	1	1	1	1	1	1
89460	PNUSAC007689	USA	22	ST-22	1	1	1	1	1	1	1	0
89481	PNUSAC007396	USA	22	ST-22	1	1	1	1	1	1	1	1
89632	PNUSAC007416	USA	6631	ST-22	1	1	1	1	1	1	1	1
89663	PNUSAC007250	USA	22	ST-22	1	1	1	1	1	1	1	1

89688	PNUSAC006832	USA	22	ST-22	1	1	1	1	1	1	1	1
89716	PNUSAC007224	USA	22	ST-22	1	1	1	1	1	1	1	1
89785	PNUSAC007058	USA	22	ST-22	1	1	1	1	1	1	1	1
89883	PNUSAC006919	USA	22	ST-22	1	1	1	1	1	1	1	1
89925	PNUSAC006530	USA	22	ST-22	1	1	1	1	1	1	1	1
90040	PNUSAC006461	USA	22	ST-22	1	1	1	1	1	1	1	1
90098	PNUSAC006352	USA	22	ST-22	1	1	1	1	1	1	1	0
90176	PNUSAC006185	USA	22	ST-22	1	1	1	1	1	1	1	1
90195	PNUSAC006208	USA	22	ST-22	1	1	1	1	1	1	1	1
90218	PNUSAC006126	USA	22	ST-22	1	1	1	1	1	1	1	1
90268	PNUSAC006229	USA	22	ST-22	1	1	1	1	1	1	1	0
90416	PNUSAC006494	USA	22	ST-22	1	1	1	1	1	1	1	1
90434	PNUSAC006634	USA	22	ST-22	1	1	1	1	1	1	1	1
90468	PNUSAC006710	USA	22	ST-22	1	1	1	1	1	1	1	1
91162	FSIS11813599	USA	22	ST-22	1	1	1	1	1	1	1	1
92236	FSIS1700038	USA	22	ST-22	1	1	1	1	1	1	1	1
92485	FSIS1609192	USA	22	ST-22	1	1	1	1	1	1	1	1
92855	FSIS1702182	USA	22	ST-22	1	1	1	1	1	1	1	1
93382	FSIS11807311	USA	6631	ST-22	1	1	1	1	1	1	1	1
93992	FSIS1607631	USA	22	ST-22	1	1	1	1	1	1	1	1
94560	FSIS11917465	USA	22	ST-22	1	1	1	1	1	1	1	0
96042	19OR02CB17-C1	USA	22	ST-22	1	1	1	1	1	1	1	1
96043	19OR02CB08-C1	USA	22	ST-22	1	1	1	1	1	1	1	1
96168	CVM N17C743	USA	22	ST-22	1	1	1	1	1	1	1	1
96188	CVM N17C700	USA	22	ST-22	1	1	1	1	1	1	1	1
96729	CVM N17C221	USA	22	ST-22	1	1	1	1	1	1	1	1
97084	CVM N16C571	USA	22	ST-22	1	1	1	1	1	1	1	1
97344	CVM N18C229	USA	22	ST-22	1	1	1	1	1	1	1	1
97485	CVM N16C145	USA	22	ST-22	1	1	1	1	1	1	1	1
97558	CVM N16C572	USA	22	ST-22	1	1	1	1	1	1	1	1
97669	CVM N16C392	USA	22	ST-22	1	1	1	1	1	1	1	1
97678	CVM N16C564	USA	22	ST-22	1	1	1	1	1	1	1	1
98137	CVM N18C230	USA	22	ST-22	1	1	1	1	1	1	1	1
98298	CVM N18C133	USA	22	ST-22	1	1	1	1	1	1	1	1
98471	FSIS11921517	USA	22	ST-22	1	1	1	1	1	1	1	0
98593	FSIS11921108	USA	22	ST-22	1	1	1	1	1	1	1	1
99786	FSIS1606361	USA	22	ST-22	1	1	1	1	1	1	1	1
101724	FSIS11920246	USA	22	ST-22	1	1	1	1	1	1	1	1
103049	FSIS11705015	USA	22	ST-22	1	1	1	1	1	1	1	1
103366	FSIS11807811	USA	22	ST-22	1	1	1	1	1	1	1	1
103802	FSIS11704019	USA	22	ST-22	1	1	1	1	1	1	0	1

103991	FSIS1701165	USA		22	ST-22	1	1	1	1	1	1	1	1
103996	FSIS1701167	USA		22	ST-22	1	1	1	1	1	1	1	0
104139	FSIS1710050	USA		22	ST-22	1	1	1	1	1	1	1	1
104231	FSIS1607419	USA		22	ST-22	1	1	1	1	1	1	1	1
104522	FSIS11918782	USA		22	ST-22	1	1	1	1	1	1	1	1
104930	FSIS11816898	USA		22	ST-22	1	1	1	1	1	1	1	1
105352	FSIS11814778	USA		22	ST-22	1	1	1	1	1	1	1	1
108825	9.17H	Estonia	2017	22	ST-22	1	1	1	1	1	1	1	1
108904	KKC291	USA	2018	22	ST-22	1	1	1	1	1	1	1	1
108907	KKC294	USA	2018	22	ST-22	1	1	1	1	1	1	1	1
108908	KKC295	USA	2018	22	ST-22	1	1	1	1	1	1	1	1
108910	KKC299	USA	2019	22	ST-22	1	1	1	1	1	1	1	1
108913	KKC302	USA	2018	22	ST-22	1	1	1	1	1	1	1	1
					Total present	287	287	286	287	281	287	286	274
					Percentage (%)	100	100	99.7	100	97.9	100	99.7	95.5

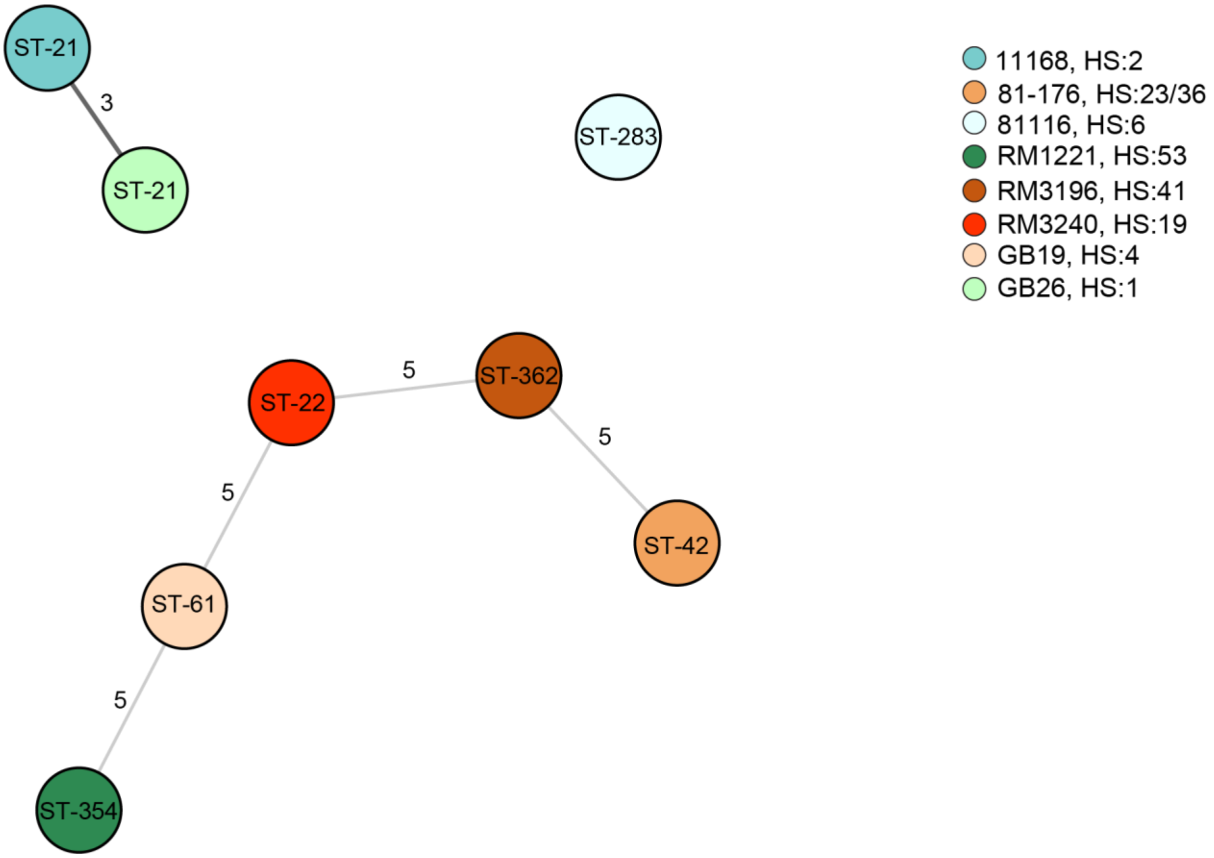

Supplemental figure 1. Minimal spanning tree of *C. jejuni* strains with unrelated capsular Penner serotypes and genetically diverse genetic backgrounds based on multi locus sequence typing (MLST) clonal complexes. The 3 and 5 indicate the number of MLST alleles that were different (7 genes MLST).

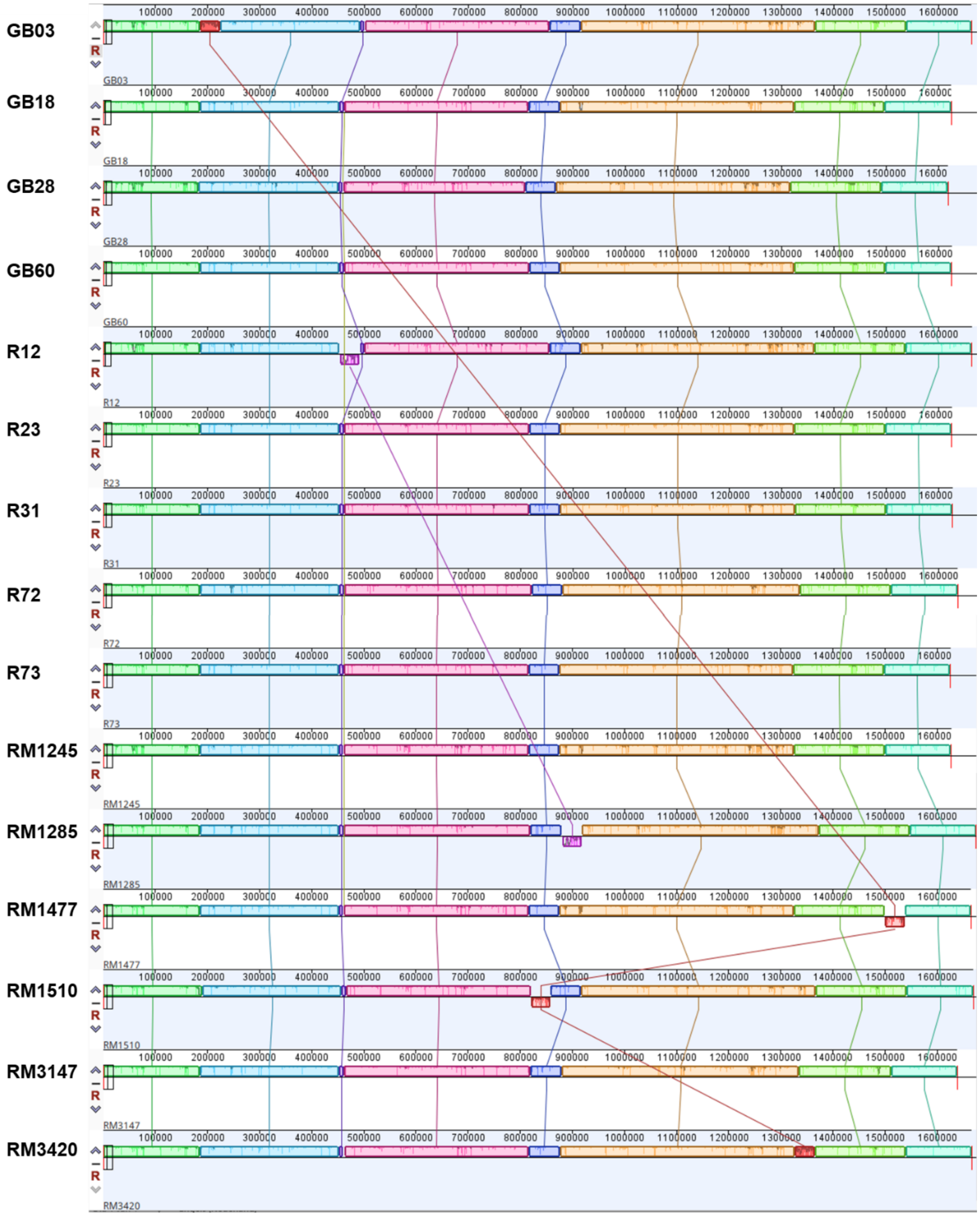

Supplemental figure 2. The presence of *Campylobacter jejuni* integrated elements (CJIE)s in the genomes of *C. jejuni* HS:19 strains. To identify large gene insertions and gene rearrangements in the genomes, a Mauve alignment was performed on the complete genome sequences. The CJIEs are indicated in red and violet and connected with a red and violet line.

A.

B.

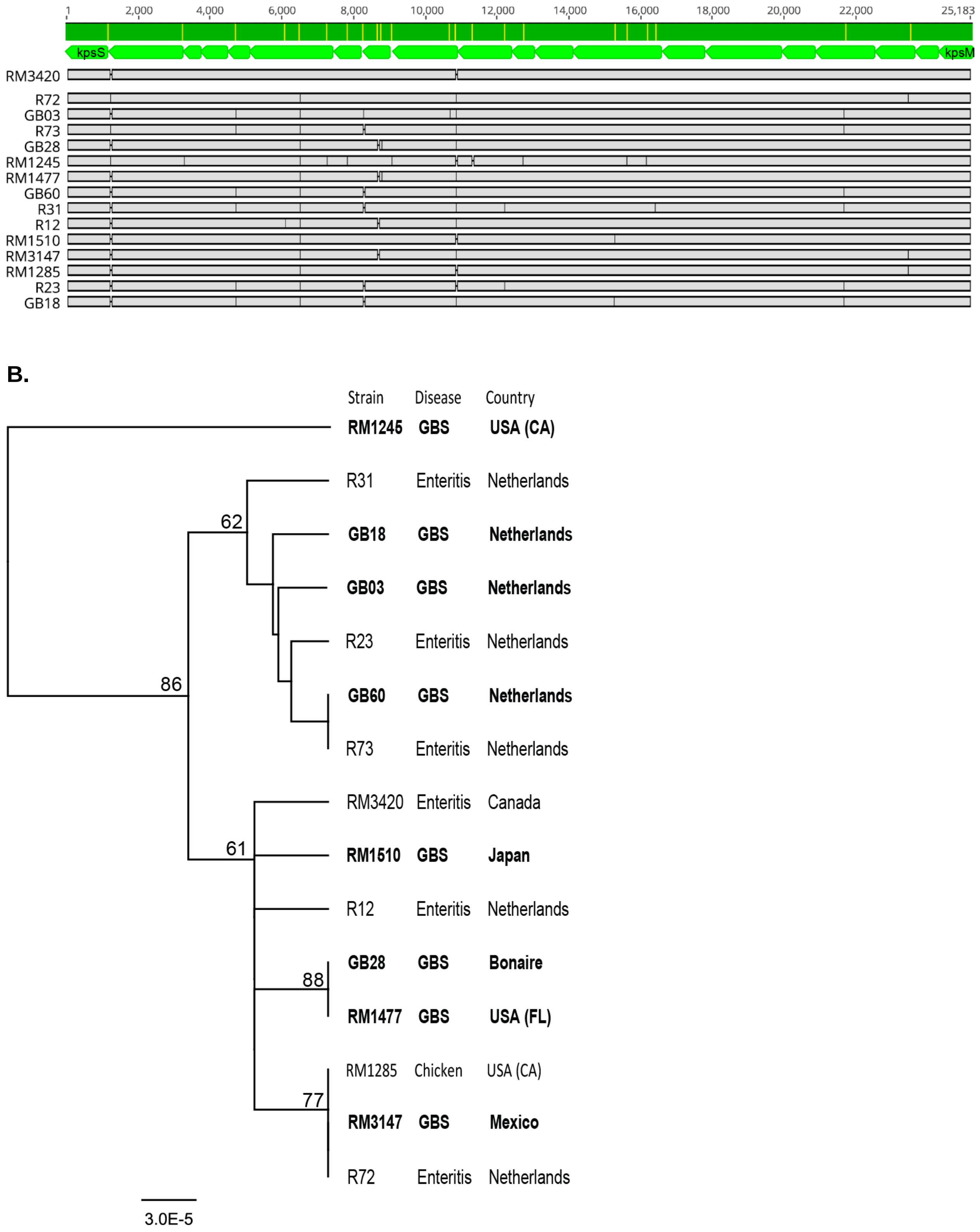
1
2,000
4,000
6,000
8,000
10,000
12,000
14,000
16,000
18,000
20,000
22,000
25,183
kpsS
kpsM
RM3420
R72
GB03
R73
GB28
RM1245
RM1477
GB60
R31
R12
RM1510
RM3147
RM1285
R23
GB18
Strain Disease Country
RM1245 GBS USA (CA)
R31 Enteritis Netherlands
GB18 GBS Netherlands
GB03 GBS Netherlands
R23 Enteritis Netherlands
GB60 GBS Netherlands
R73 Enteritis Netherlands
RM3420 Enteritis Canada
RM1510 GBS Japan
R12 Enteritis Netherlands
GB28 GBS Bonaire
RM1477 GBS USA (FL)
RM1285 Chicken USA (CA)
RM3147 GBS Mexico
R72 Enteritis Netherlands
62
86
61
88
77
3.0E-5

Supplemental figure 3. Alignment and phylogenetic tree of the capsule biosynthesis locus of *C. jejuni* HS:19 strains. MUSCLE alignment (A) and UPGMA clustering (B) and of the capsule biosynthesis locus (genes *kpsS* - *kpsM*) of *C. jejuni* HS:19 strains. The sequence of the capsule biosynthesis locus was highly conserved in all strains with a maximum of 10 SNPs difference for RM1245 when compared to the reference RM3420 and a minimum of 2 SNPs for strain RM1510. UPGMA clustering of the sequence of the capsule biosynthesis locus did not lead to a separation of GBS- from enteritis-related strains

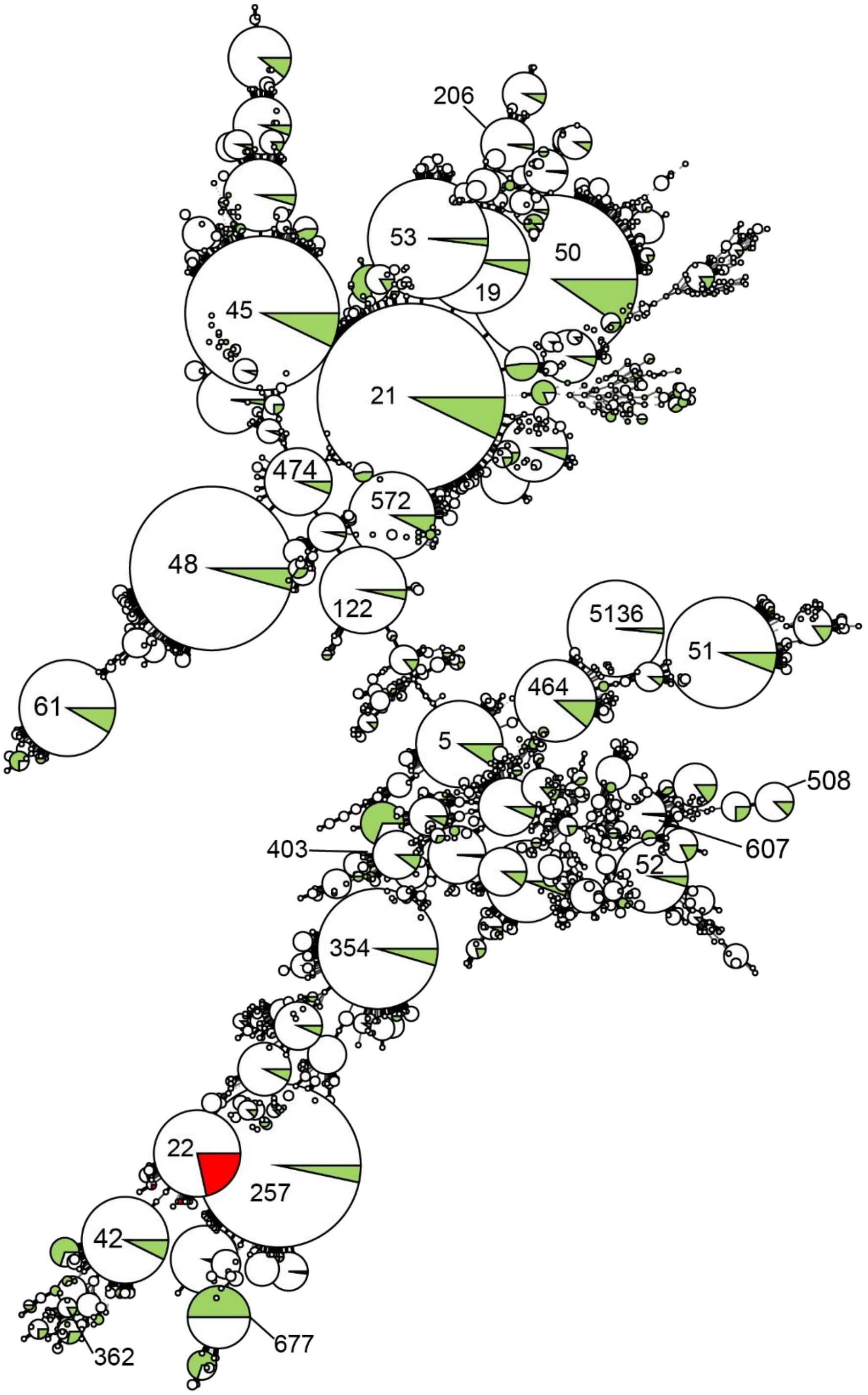

MLST sequence type or clonal complex 22 from this study (n = 36)
Other MLST sequence types from this study (n = 874)
MLST sequence types from pubMLST (n = 10,000)

Supplemental figure 4. Minimal spanning tree of multi locus sequence type (MLST) data from this study and PubMLST. Comparison of multi locus sequence types of the sequenced HS:19 strains and the downloaded genomes from this study vs. a randomly downloaded subset from PubMLST.

The MLST sequence types of the sequenced HS:19 strains and the downloaded genomes from NCBI plotted together with a randomly downloaded subset of PubMLST sequence types.

Correlation MLST sequence type; this studt vs PubMLST = 0.771, $p < 0.0001$ (Pearson).

Correlation MLST clonal complexes; this study vs PubMLST = 0.901, $p < 0.0001$ (Pearson).

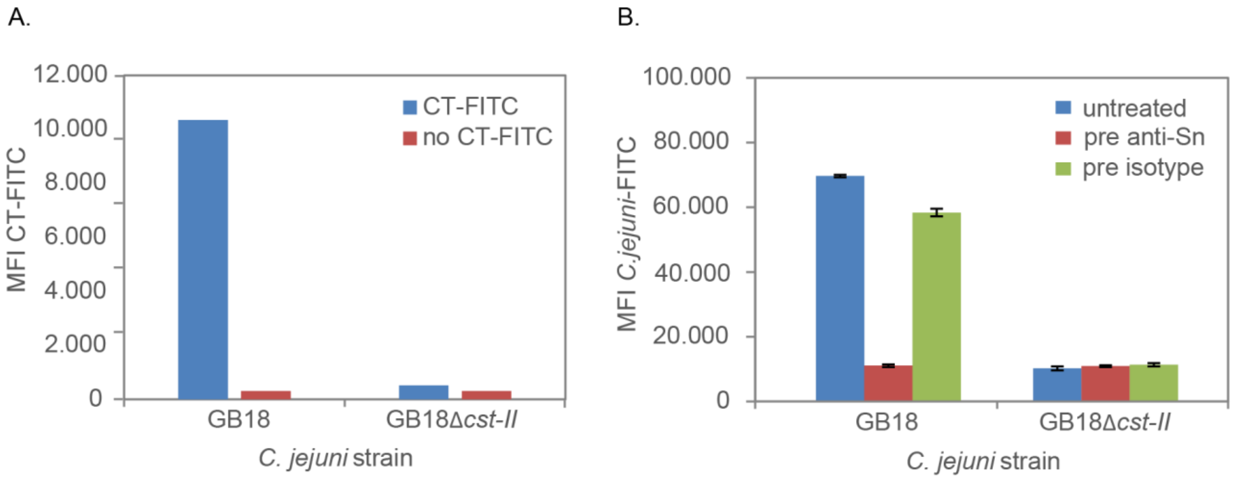

Supplemental figure 5. Binding of a *C. jejuni* HS:19 wild type and knockout strain to cholera toxin and sialoadhesin. A *cst-II* knockout mutant was generated in the *C. jejuni* HS:19 strain GB18 through a natural transformation using DNA isolated from a previously generated *C. jejuni* knockout mutant strain GB11Δ*cst-II* (5). The GB18 wild type and knockout strain were incubated with FITC-labelled cholera toxin (CT-FITC) (A) or with sialoadhesin-expressing THP-1 cells (THP-1-Sn) (B) as described (1), and the binding was measured with FACS. Pre-anti-Sn, pre-treated with an antibody directed to Sn; pre isotype, pre-treated with an isotype control antibody.

Reference:

1. **Heikema AP, Koning RI, Duarte dos Santos Rico S, Rempel H, Jacobs BC et al.** Enhanced, sialoadhesin-dependent uptake of Guillain-Barre syndrome-associated Campylobacter jejuni strains by human macrophages. *Infect Immun* 2013;81(6):2095-2103.

Supplemental Data.

Supplemental Data 1. Sequence data relevant for this study.

>HS19 Cluster I Campylobacter jejuni subsp. jejuni str. RM3420
ATGCTAGAACTTATCACACAAAAAGATCAAGATTTTTTGCAAAATATTGAAAACCTTGATGAAGAATATATTCAAAAATT
TATTAATAAAAAAATCAGTGAAATAGCTATAGCTATTGAAACAGCAGCAGAGAATGCTGATAAAGCAAAAGATAGAACAC
AAAAAGCCAAAAATCTTAATACAGATAGTGATTGGCAAACTTATATACCAATTTTTGGACGTTGGCTTGGAGAAACATCA
GAAGAAAAAAAAGAAATTAAATCCAATATGATCCTTGAAGTAGCAGAGTTACAGAATGAATCAATGAATCAAATGACAAC
AATTCTAAAAGAAGTTGTAATATTTTTTACTTCCAGTTTTTGTATTGCTACGCGTATGAATCAAGCTTTATCTCTTATTA
TTGCGCAAGGTTTTGTAAAGAGCGATGGCAAGGTAATTCGTTTATCTAAAGCCGCTAAAGAACAATTTCAACAAATACAA
AAGTTCACACTGAGTTTTATAGAAGATCATGAAAAACACAAAGATACGATAAATAATATCCAAGTAGAATTAGATAAAAA
AAATCAAATTGATGATGAGCAATATAAACTCATAGAAAAACATTATCAAGAATTTATACAATATAAAAATTATAATGACA
AAATTGTTCAAGAGCAAGAATGTAAAATCAATGAACTGAAAGATATTTTAAATAAAAGAAAAAATGTTTTTATAAATAGC
ATTTCAATTTTAGCTTTGATAGTTTCTGTTGCGTCGATTGTATTATATTTTATAGGGCGTTAATTTATGAATTTTTTAAG
TTCTTTAAAAGATAAGGCGGTAAATGCTTCAGAAGCAATTAAAGACAAAACTATTAAGACTGCTGAAGTTGTTAAAGATA
TTGGAATGGAAGTAAAATGTGGGATTGGTTGGCACGCGGGTGAATATCAAAATGAAAAAGATAAACCAAAATGTTTTTTT
TCCAAAATTTGCCCTGATTGTGGAAAATATCTTACAAAAAATCAACATGATTTTGAAGCTCCTGAAATATTAAATCCTGA
TAATTGTTATGGATATAGACGATGTACTCTTTGTTCTATTCAAGTGTTTGATAATTTTCATAATTATTATGAAATCAAAA
AAGATTCAAAATGCAGAATGCACGAAAAATGTAATTTATGCGGACATGAAAGATTAGGACAAACAAGACACAATTGGAAA
TACGACGAAAGCGGGCAAAAAATTTGTCTAGATTGCAAAGAAACAGTATAA

>HS19 Cluster II Campylobacter jejuni subsp. jejuni str. RM3420
ATGAAAAATTTCAAAGATAGTGGCATAGAATGGCTTGGGGAAATTCCGGAGCATTGGAAGTTGATTAAGTGTAAAAATTT
TTTTGTTTTAAAGAGTATACCTATAGGTGATTTGTGGAATAAAACAAAATTACTATCATTAACTTTAAATGGAGTTATAG
AAAGAGATATTAATAATCCGGAAGGAAAATTTCCAAGTGATTTTTCTACTTATCAGATAGTTAAAGAAGGCGATTTAATT
TTTTGTCTTTTTGATGTAGCAGAAACCCCTAGAACTATAGGATTATCAAAATTAAATGGAATGATTACTAGTGCTTATAC
TATATTTGAGATTAAAAATCAAGAAAAACGATTTTTAGAATATTTCTTTATTGACCTTGATAACAGAAAAAATCTTAAAT
TTTTATATAGAGGTTTAAGAAATACAATATCAAAAGAAGATTTACTGAATCTTAAAATTCCACTTCCACCACTAAAAGAG
CAAGAACAAATCGCAAATTTTTTAGATGAAAAATGCGAGCAAATTAAAAATTTCATAGAGAAAAAAGAAAAACTCATCAC
ACTTTTAAAAGAACAAAAACAAGCCTTTATAAACAAAGCAACCACAAAAGGACTTGATAAAAATGTAAATTTCAAAGATA
GTGGCATAGAATATCTCGGAGAAATTCCACAGCATTGGAAATTGGTAAGACTTGGGTTAATATTAAAAACAAGTAGTGGC
ACTACTCCAGATAGTGGTAATGATAAATATTACAAAGGAGGACAAATTGTTTGGATTAACTCAGGAGATTTAAATGATGG
ATTTTTGAAAGATAGTAAGAGAAAAATAACTCAAGATGCTCTTGATGATTATTCGGTTTTAAAAATTTTTGATAAAGATT
CTTTAATTATAGCAATGTATGGAGCAACTATTGGAAAAACGGCTATATTAAAAGTGAATGCCTGTGTTAATCAAGCTTGT
TGTGTATTGGAAAAAAGTGCTTGGTATAATACTTTTTATTTGTTTTATTTATTTAATAGATATAAAAAAGAACTTATTTC
AATGGGAAGTGGAGGCGGACAACCAAATATTAGTCAAGATATAATTAAAAATCTTAAAATTCCACTTCCACCACTAAAAG
AGCAAGAACAAATCGCAAATTTTTTAGATGAAAAATGTAAAAAAATAGATTTATTGATAGAAAAAACCGAAAAACAAATA
AAACTCATAAAAGAATACAAAACCACACTCACAAACCAAGCGGTTTGTGGTAGGATAGGAAAAAGTTAAAATAACTATAA
ATTTGGAGGTTAAATTATGAATAAATACACTAAGCCATGTTTTAAAGGAGCGGCATTTAATTGTCCTCATTGTGGAGTAT
ATTCGCGTATGGAGTGGGATGGTTTTTATAATGGGTATAATAATAGGATAAAACAAGTAGAAGGATATTCTTTTTCTGAA
TCTACTTGTTATCATTGTGAAAGGAGTGTTATTTGGTATTTAAAAGATGAAAATCCAAAAATATTTTTTCCAAGGGAAGT
TGCCATTCCTCCTGAAGAAAATATGCCTGAAAATGTTAAAGAAATTTATGAAGAAGCATCTTTAGTTTTAGGTGACTCTC
CTAGAGCTTCATGTGCTTTATTGCGTCTAGCCTTGCAAGAATTAATGAAGTATCTTAAAGAAAATATTCAAATATATAAT
GGATTAAAAAAATAGAAATATTAATGAAGATATCAAAGAAATTATAAATATTGGAAATTTTTATCAAGAACAAAAAGAGAG
TTTAGAAGAAGCTATGAATTCTATCAGGTTGATTGGCAATAAGGCTTCTCACCCTTCTGAATTAGATATTAATGATAATT
CAGAAATTGCAAATATTCTTTTTGAAATGATTAATTTTATCGTAGGAGAAATTATTACAAAACCAAAAGAGAGGGAAGAA
AGATTAAATAAATTAAAAAGTGCTATAGGTGAAAAACAATAAATCATAAGGAAAAATATGGAAAATAAACTATTTGAATA
CGATGAAGTTTTAAAACAAACAGATGAAAAAAGACACCTTCTTTTAGGAAATGGTTTTAGTATGGCTTATGATAAAAATC
GCTTTTCATTTACAAGTTTGTTGCAAAGTGCTATTGATAATGGTATTATAGAAGAAAATTCAAATATTCATAAAATATTT
AAAAATAACAATACTAGTGATTTTGAAGAAGTTGTTAAAATCTTAGAAAATACTTCTAAAATTTTAAAAATTTATACTCA
AGATGAAAGATTATGTGAGCAATTATCGAACGATTCTGAAAAATTAAAAAATTTTTTAGTTGATATTATTACAAACAATC
ATCCAAATAAAATACAAAATCAAAAAAAAATTAAACTCAACAGCTCAATTTATAGAAAATTATCAAAAAATATTTACATTA
AATTATGATTTATTATTATATTGGGTTATTCATAAAATAATGCAAAATAGAAAAGATTTTAAAGATGGTTTTGGTGGTAA
TGATGGAGAATATGTTGTATTTGATGAAAGTAAAAAAGATATAACTTGTTTTTATTTGCATGGTGCTTTACATATTTTTG
ATAATGGTAATAAAATAATTAAAAAAACTTATTCAAGAACAAAAAAGCCCTTAAAAGAACAAATTACAGAAGAACTTAAC
AATAATAGATATCCAGTTTTTGTATCGGAAGGCACATCAGAACAGAAAAAAGCAAAAATTATTCACAACGCATATCTAAA
CCATTGCTATAAAAGTTTATCTTATATAGATGGAGATTTAATTGTTTTTGGAACAATGTTAAAATCAAACGATGAACATA
TACAAGATGCTATATTAAAAAGTAAAGTTAAAAATATATATTTTGGTGCATCAAGTTTAGAAAAAGGTAAAAATGACTTA
AATAGTTTTATTGAAAAAAAATAATAATTTAGAAAAAAATAAAAAGCAAATTTTCTTTTACGATTATAAAAGTGTAAAAAT
TTGGTAA

>HS19 Cluster III Campylobacter jejuni subsp. jejuni str. RM3420
ATGAATAAAGATCTTTTTTTAAAAAACACTCAAGCTTTGTTTGAAGTTGATCAAATTTTAGCGTATAAATTACGTGCTTT
AGAAAAAAATGATTTTAAAATACTGCAAAATGAAAATGGAATCAATTTTATAAAAGATGATATCATGCTTTATAAAAATC
CCGAACAAGAACTTTTAGAAAATTTAACTCTTTTTCAAAATAAGTATAAAAAATATCCTGTATTATTTTTCTACGGTTTT
GGAAATGGTATGTTTTATAAAACCTTATGTGAAAATAAAAACCATAAACACATCATAGTTTTTGAAGATGAGTTAGAAAT
TTTAGCTCTAGCTTTACATCTTTTTGATTTTAGCAAAGAATTAAAAAATGAAAAATTAATACTTTTTCATACTCCAAATA
TCACCACTGCACAACTTACGACACTTTTTATGTACGAACACATACAAAAAAGTGTGAAAATTTTTAATCTTTTTATACAT

AGTGATTTTTATCTTAAATTCTATTCTCATCAGATACAAGAACTTAATAAAAAACTTATTGAAAATATTCGTTTTATTGT
TTTAGCTAAAGGAAATGATCCTTATGATTCTATCATCGGTATCAAACACATGCTTAATAATCTTCCTAAACTTTTAAACC
ATGGTGTTTTTCAAAATTTCCTAAAAGCAAGAAAACAAAAAGTTAAAAATGCCATCATTGTTTCAACAGGACCTAGCTTA
ACCAAACAATTGCCTTTACTAAAAAAGTATGCAAACAAAGCCACTATTTTTTGTGCTGATAGTGCTTATCCTATTTTAGC
AAAACATAATATAAAACCTGATTATGTTTGTATGTTAGAAAGAGATGATATAGTTTCTAAGTGTTTTGATAATGATTTTA
AAGAATTTGATCAAGGAATTTTATTTATCATAAGCTCTGTGGTACATCAAGAAGTTATTGATTTTCTTGAAAAGAACTCT
AGAAAATATATACTAGTACATCGCCCTTTGCATTTTGCTGTATCTTTAAATCTTAAAGAATTTGGTTATATTGGGGTTGG
AGCGAGTGTTGCAAATATGGCTTATGAATTAGCTGCAAGTTTAAGACATGAAAATATCATTTTAATAGGTCAAGATCTTG
CCTATGCTAAAGATGGAAGTTCACATCCTAAAGAACATATTTTTGGAAATGAAGGAGAAAAAATACGCGGTGAAGTTTAT
ACGCTTGCTTATGGGGGAGAAAAACAAGTAAGAACTCAACTCACTTGGAACTTGTTCCGACAAGCATTTGAAAAAGATAT
TTTTTGGGCAAAAGAAAAATTAAAAATCAACACTTATAATTGCACCGAAGGGGGTGCTAGGATAGAAGGAACCATAGAAA
AACCCTTTAAAGAAATGTGTGAAACTTTACTTAAAGAAGATCTAAAAAAACCTTTTGATCTACCTAAAATTTTAGAAAAC
AATGAGACAAAAAATAAATTTTTACAAACTCAAAAACTGCTAATTAAAAATATTGAACAAAGTGAAAAATTTATAAAGAA
ATGTCAAAATGAACTAAAAAAACTTGACTTTGAACTTGGCAAATCTCAACTTAATTTACAAACTCTAATAAAAATTAAGC
AAAATCTTCTAATTTTTTTTAAGGATTTTAAAAGATTAAAACTTTTCAATGAGCTCACTCAAGCCATATATTATCACAAC
GAATGCGAAATTATGCATTACGAAGTTTTAAATGATTTAGAACAAAATGAAAAAATTAAAGACTTTTTAACCAGTCAAAA
AACTTGGTGGTTGCAAAGCTTTGAGTATTTAAATACTCAAAACCAAATCATAAAAGAAGCTTTAAAAAAATACAAAAATG
ACGATATTTCATAAATAAAAGGAAAAATTATGAAATTTAATCAAAATCAAATTAAACTTTTTAATAGAAATATCAATGCT
TTAAACAATACTGTTTTAAAGGAAAACTTAAAGCAAATTAAATCAAGTAAATTTAAACTTATTTTAGGTAAAGATAATCT
TGATATAAATTTAAAAAATACAAGTGATAATACCTTTCTTTATGAAAATGCTATAGATGAATTAAACTCCATGCTTAATA
TTTATAATGACAAATATCTACTTTATCCTGTATTGTATTTTTATGGTTTTGGAAATGGGATTTTATTTAAAGCCTTACTT
CAAAATAAAAATCACCAGCATATAGTTGTTTTTGAAAAAGAATTAGAAATTATCAAGATCATGCTTCATCTTATGGATTT
TTCACAAGAATTAAAAGAAAATAAACTTATAATACTTGATGTTAACTCCTTAGAATTTCAAGATTATTATGACTTATGTT
CAAGCAATCCCTTTTTTGGTTTTTCAAGAACTTATTTTCTAGAACTTAGTAGCGATTATTATGAAAAAGATAAAGAAGAA
ATATTAAATTTAAATAATAATCTTATAGATAAATTTAAAAATGCTATATTGTCAAGTGGCAATGATTCTAAAGATGTTTT
ACAAGGCATAGAACAACTTATCTACAATCTTCCTAAAATGATCACTCATACTGCTTATCAAGATTTGCTCAAAAAAAGAG
AAAATCTTAGTGATACTGCTATTATCGTTTCAACAGGACCTTCTCTTACCAAACAACTTCCTATTTTAAAAAAATATGCA
AATAAAGCAACAATCATATCAGCAGATTCTTCATATCCTATTTTAGCAAAGCATGATATCAAACCTGATTATGTTGTATC
TTTAGAAAGAATTTTATTAACCTCAGAATTTTTCAATAACAATTTTGGTGATTTTGATAAAGATATTTTATTTATCACAA
CTCATTTAACCCATCCTCAAACTATAAAAAATTTAGAAAAAAATAATAGAAATTTTATGTTAGCTTATAGGGGTTCTGAA
TTTATAAAATACTTAAAAATTAAAAATTTTGGAGAACTATCTGAAGGACATAGTGTAGCAAATGTAGCTTATGGACTAGC
TGTATTTTTAAGACACAAAAATATTATTTTCATAGGACAAGATTTAGCTTATTCTGATGATGGATTTTCTCATACAAAAG
ATTATAGAAATCTTAACAAACATGAAGGTCATTATGAAAGAGACTTTGGACATTTTAGAACCATAGCTTATGGGGGAGTA
GGTTTTGTGGAAAGCTCTTTTTATTGGAGTTTATTTAGATTTTTTTTAGAAAAAAGAATTTATATAACCAACCAATCTGG
GTTTTGTAATACTTATAATTGCACCGAAGGGGGTGCTAGGATAGAAGGAACCATAGAAAAACCCTTTAAAGAAATGTGTG
AAACTTTACTTAAAAACAATTTAAATAAAAATTTTCCTAAAATAGTTCCATTGAGCAGTCATAAGCAAAATGAACTTTTA
TTAAAATGTTATTACAAGATTATAAAAAGTATAAATCACTGCAAAACTTTTAAAAAAGAACTTTTAAAATCTTATAATCA
CATACAAGAAAGTTTTTCAAATCTAAATTTAATTTCAAATTTAGATGAAGGTAAAGAAATATTAAACTATCTTATCCAAG
AAATAGACAAAACAAAATTCAAACTTGAAGATGAAAAAATGCTAGACTTATATGAAATTTTAAATCCAATTCTAACTCAA
TTTGAACTTAATTTAGCAAGAATTTATGTTTTAAACCCAAAAACTTCAGAAGATTCTTATAATAAATCTTTACTGTGGGT
TAAAGAACATATAGAATTTTTTAAAATGATTTATACTCACATAAAAGCACAAGAAAAAGCTTTGATAAAAAATATCACTC
CTTTAGAAAATGAATTAACTCAAAGAAATTTAGAAAAATACAAAAGGAAAATAAATGCAAAATACAATTTTTAACAAAAA
TTTAAAAGCCATGAATGGCAAGGAATACAATGAACTTAAAGAAAAATTAGTCAAAATAAAAGAACTTAGAGAATTTTCTT
ATACTTTTGGAAAAGATAATTTAGATATTAATATCATTCAAAAAAGAAATTTAAAAACACTTTATAAAAATCCTTTAAAA
GAGTTAGAAGAAAAAAATTGAATTTTTTAAAAATTATGAAAGATATCCGGCTTTATTTTTTTACGGTCTTGGAAATGGAAT
TTTATATAAAGCTTTATTTCAAAATGAAAATCATAAAAGAATAGTTATATTTGAAAAAGAAATTGAAATTATTTTTATAG
TTTTAAATTTATTAGACTTCAGCGAAGATATAAGAAAAGGTAGATTTATTTTAATCCATACTCCAGAAATGACTTATGCA
AAAGCAGATATTATTTTTTCTTTAAGTCCTATTGATCGCTTTTTTAAAACTTATAATTTACATTTACATTCTGATTTTTA
TAAAAACTACAAAGACGATACTTTAAAAATTAATACTTTAAATTTAAAGGCTATTAAAAACATTAGTTTAAAAAAAGGCA
ATGATCCTAAAGATGCTATGCAAGGGATCGAACAATTTGTAAGAAATATCCCAAAAATGATCTCTCATCCAAGCTATCAA
ACCTTGCTTAAAAAAAGAAGAAATTCCAAAAATGAAAATGTAGCCATTATAGTTTCTACAGGACCTAGTCTTGAAAAACA
ACTTCCCTTACTTAAACAATATACAAATAAAGCAACTATTTTTTGCGCTGATAGTGCTTATGCCATACTAGCAAAACATA
ACATAAAGCCTGATTATGTTTGTATGATGGAAAGAGATGACATCACAGCAGAATGTTTTAATAATGATTTTAAAGATTTT
GATCAAGGAATCACTTTTATTGTAACTTCTTTAGTACACAAAAATACAATTTCTTATCTTGAAAAAAACAAAAGATCTTA
TATCTTGGCAACAAGATTCTTACCTTTTGCTAGATCAATAGATCTTAAAGAATTTGGATACATAGGCGGTGGGTTAAGTG
TAGCCCATATGAACTATGAACTAGCTATCAATCTAAACTACAAAAACATTATTTTAATAGGACAAGATCTAGCTTACGCA
AAAGATGGAAGCTCTCATTCAAAAGGCTTTATCCACGAAAAACTCCATGATGGACACCATCAAAGAGATTTTAACAAATA
TACAACTATAGCTTATGGGGGGAAAGGAACAGTTCAAAGCTCTGAAGTTTGGACTATATTTAGAGAAATTTTTGAAAGCT
TTATAGCATCAAATAAAGACAAAATAAAAACTTATAATTGTACTGAAGGTGGAGCTAGGATAGAAGGAGCTATAGAAAAA
CCATTTAAAGAAATGTGTGAAATTTTGCTTAAAGAAGATCTTAAAAAACCTTTTGTTAAAATTCCTAAACTTTCTAAAAT
TCAAAGAAACACCTTGATGCTAAAAGCCTATACAAAAATCAAAAAACAAATTCTTTTAGCTAATACTTTTTTAAAAGAAT
GTAAAAAAATCAAAAACCAATTAGCCTCTTTAAAAAGAGAAAAATCTAAACTTACTCTAGATCAAATCAATCAAAATCTA
GATAAATTTAAAACTCGTTTAGATTCCAATCGCTATAATTTTTTGTTTGAAATTCTAGGTCCAACACTTCATCATGAAGA
AGGTATTTTATCACCTTTGTATGTGCAAAGCATTCAAAATGAAAGCGAAAAACAAAATAAACTTTTTGCTTGGCTTTACG
CTCATGAATCTTTAATCGAAAGTATTTATGATCTTACCAACACTCAAAATACCATCTTAAAAAAAGCTATCATTCCTTTG
CAAGATGAATTAGAAAAAAGAAATTTGATCTAA

>HS19 Cluster IV Campylobacter jejuni subsp. jejuni str. RM3420
ATGAACTTAGAATTTTCAAAAGAAACACAACATTTTTTAACTAATTATTGTAAAGATAACAATTTAAGTGAAAAAGAAGT
TTTAGAACTTGCTTTAAGTTATTTAGAACATAAAATAAGAATTGATGGTTATAAAAAGGATATAGAACTTTATAAGCAAG

GTAAATTAAAGACTTTAGATTTTGATGAAACTTTTAATGATATTAGAAAAGATTTAGAATAAATGAAAATTGTTCCAACT
CCTAAATTTAAAAATGAGCTTAGGCATATTGTTAATTTTATAAACTTAGATAGTTCTTTTTATGCTAAAGAATTTCTTAA
TGATATTTATTCAATTTGTAGAAATCTTTCTTTTATGCCTTATAAAAATAGAAAATCTTTGTCTTTTGATAATGAAAATA
TAAGAGATTTGATTTTTAAAGGTTATATTATCCCTTATCTTATCGATAAAAGTAAAAATGAAATTGTGATTTTAGGCATA
TATAAAAGTAATCTTTGGGATTGA

>HS19 Cluster VI Campylobacter jejuni subsp. jejuni str. RM3420
ATGGAAAATGAACTCATTGTTAGTAAAAATATGCAAAATATAATCATAGCAGGAAATGGACCTAGCCTAAAAAATATTAA
TTATAAAAGACTGCCTAGAGAATATGATGTTTTTAGGTGTAACCAGTTTTATTTTGAAGATAAGTATTATTTAGGAAAAA
AGATTAAAGCAGTATTTTTTAATCCTGGTGTCTTTTTACAACAGTATCACACTGCAAAACAACTTATACTAAAAAATGAG
TATGAAATAAAAAATATTTTTTGCTCTACATTTAATTTACCTTTTATTGAAAGCAATGATTTTTTACATCAATTTTATAA
TTTTTTCCCCGATGCAAAACTTGGCTATGAAGTTATTGAAAACCTTAAAGAATTTTATGCTTATATAAAATACAATGAAA
TTTATTTCAATAAAAGAATTACTTCGGGCGTCTATATGTGTGCAATTGCTATTGCATTAGGATATAAAACCATCTATTTA
TGTGGCATTGATTTTTATGAAGGAGATGTTATTTATCCTTTTGAAGCTATGAGTACAAATATAAAAACAATCTTTCCTGG
AATAAAAGATTTCAAACCTTCAAATTGTCATTCTAAGGAATACGATATAGAAGCATTAAAATTGTTAAAATCAATATACA
AAGTTAATATCTACGCATTGTGTGATGATTCTATTTTGGCAAATCATTTTCCTTTATCAATTAATATTAATAACAATTTC
ACTTTAGAAAATAAGCATAATAATTCTATAAATGATATTTTATTGACTGATAATACTCCTGGCGTAAGTTTTTATAAAAA
TCAACTTAAAGCTGATAATAAAATTATGCTTAATTTTTATAATATTCTTCATTCTAAAGATACTTTAATTAAATTTTTAA
ACAAAGAAATTGCGGTATTAAAAAAACAAACCACTCAACGAGCTAAAGCAAGAATCCAAAACCATCTATCCTATAAACTA
GGACAAGCTTTGATTATAAATTCTAAAAGTGTATTAGGTTTTTTATCTTTACCTTTTATAATATTAAGTATCGTTATTTC
ACATAAACAAGAACAAAAGGCTTATAAATTTAAAGTAAAGAAAAATCCAAATTTAGCTTTACCTCCTTTAGAAACTTATC
CTGATTATAATGAAGCTTTAAAAGAAAAAGAATGTTTTACTTATAAATTAGGAGAAGAATTTATAAAAGCTGGTAAGAAT
TGGTATGGGGAGGGGTATATCAAATTTATATTCAAAGATGTTCCTAGGTTGAAGAGAGAGTTTGAGAAAGGGGAATAAAT
GATTTTCAATCTTACCCATTTAAGACAACTTGAAAATGAGTCTATTTTCATAATGCGTGAAGTAATATCTGAATTTGAAA
AGCCAGCTATGCTTTATAGTGTAGGTAAAGATAGTTCAGTGATGCTTCATCTTTTACAAAAAGCATTTTATCCTACCGTG
CCACCACTTCCTTTGGTGCATGTAGACACTACATGGAAATTTAAAGAAATGATAGAATTTAGAGATAAAAGAGCCAAAGA
GCTTGGTATGGAGCTTATCGTTTATCAAAATCCTAAGATTAAAGAATTAAACCTTTCTCCCTTTACACATGACTCATCTA
TGCACACTGACATCTCTAAAACTCAAGGCTTAAAACAAATGCTTGATTTATACCAATTTGATGCTGTTTTTGGCGGAGCA
AGAAGAGATGAGGAAAAATCTCGAGCAAAAGAGAGAATTTATTCCTTCCGTGATGAAAATCACACTTGGGATCCTAAAAA
TCAACGCCCAGAACTTTGGAATTTATATAATGGACGCCATAAAAAAGGAGAATCCATAAGGGTTTTTCCGCTGAGCAATT
GGACGGAACTTGACATATGGCAATATATTTATAAAGAAAACATTCCTATACCGAGCCTTTATTTTGCTAAAAAGCGTTCA
GTGATAGAGTATATGGGAGCTAAAATTTTAGTCGATGATGATAGAATGCCAAAAGAGCTTGCTAAAAATTCTAAAGAAGA
AATGGTTCGTTTTAGAACTTTGGGTTGCTATCCTCTAACCGGAGCTATAAATTCTAACGCTAGTAATGTCTTAGAAATCA
TCGAAGAACTTTTGCTTTCTAAGACAAGTGAAAGACAAGGCAGACTTATAGATACTGATGAAGAAGCAAGTATGGAAAAA
AAGAAAAAAGAGGGGTATTTTTAATGCAAACAAATATAGAAAAATATCTCCAAGAGCATGAAAATAAAGAGCTTTGTAGA
TTTATCACTTGTGGCAGTGTTGATGATGGAAAATCCACTCTTATAGGAAGATTGCTTTATGATACTAAAGCTCTTTTTAG
TGATCAATTAAGCACCTTAGAAAAAGATAGTAAAAAAATGGGGAACGCCGGAGATAAACTTGATTTTGCACTTTTAGTAG
ATGGGCTTGCAAGCGAAAGAGAACAAGGCATTACTATAGATGTGGCTTATAGATTTTTTACCAGTAATAAACGCAAATTT
ATTATAGCTGATACACCAGGGCATGAACAATACACTAGAAACATGGCCACAGGAGCAAGTACAGCAGATATTGCTATTAT
TTTAATTGATGCAAGAAAAGGGGTTTTAAAGCAGACTAAAAGGCATTCTTATATAGTGAGCTTACTTGGCATTAAAAATT
TTATCATTGCCATTAACAAAATGGATCTAGTTTCTTACGAAGAAAAAATCTTCAATAATATTTGTAAAGATTATGAAAAA
ATCATCCCTTATTTGCAAGAAGATATCCAAACACACTTTATTCCTATTTGTGCTTTAAATGGTGAAAATATCACTCAAAA
AAGTAGAAATTTATCTTGGTATAAAGGTGAAACCCTACTTGCACTTTTAGATGAGATTAAAATAAATAAAATCATTCAAA
ATGATTTTATCATGCCTGTGCAGTATGTTAATCGTCCGCATTTAAATTTTCGTAGTTTTTGCGGAAATATAGCAAGTGGT
TATGTAAAATTACAAGATGAAATTATAGTCTTGCCTTCTATGCAAAAATCTAAAATTAAAAGTATCATTACAAATGATAT
TAAAGATTTAAGAACTTTAGATGAAAATGAAATCATTCCAAGTCAAAATGAAGCTTGTGTTAATATGGCCGTAAGTTTAT
GTTTAGAAGATGAAATCGATATTTCTAGAGGCGATATTTTGGCATCTATCAACCATGATCTTAAAATGAGCAATGCTTTT
GAAGCTATGATTATATGGATGAGTCAAAAACAATTAGATCTTAATGAAAATTATCTTATAAAAAGAGCTCATAATCTTTG
CAATGTTAAATTTGAAAATATTAACTATAAAAAAGATATCAATACTTTTAAAGAAGAAAATGCCTCAAATTTAATACTTA
ATGATATTGCAAATTGTACTTTAAAACTAGATAAAAATTTAGCACTTAAAGAGTATAAAGATAATAAAACCTTAGGATCA
TTTATTATCATCGATAAATATAGCAATGAAACTTTGGCCGCAGGTATGATAATAAAAATTTTAAATTCACAACAAAGCCA
AAGAATTTACACTCAAGCTGAAATAGAACTTAACGCTTTTATAAGAAAAAACTATCCAGAATGGGGGTGTAGAAAAATAT
GAAAATCATAGTAGGTATCACGCTTTTAATTCTACTTATATTGCTCATACGCAACAAAATAAAGCCTGCTGTTTTATTTG
GAAGTTTGGCAGGATTTTACTACGCTTTAGGTTATCTTGATTTTAAAACTTGGATAAGTTCTTATACAAATGATTCTTTA
ATTTCACTCATGTTGCTACTTTTAGTTTCTATCGCTGTTGAAAAAACTATCATTATAGAATGGGCAAGTAAATTTATCAT
AGGAAAAAACTATAATCTTTCTCTTTTGAGATTAGGTGTAATTACTTGTGCGGTTTCGGCCTTTTTAAACAATACCGCAG
TAGTAGCAAGCTTTATGGGTATAGTAAAAAACAATAAATTTCAAGCTCCTTCAAAGCTTTTAATCCCTCTTTCTTATTTT
GCCATAGTAGGCGGTGTTATCACTCTTGTGGGAACTTCTACAAATTTAATCATCAATTCCTTTGTGGTACAAAATGGACT
ACCGAGTCTTAAAATGTTTGATTTTTTTTATATAGGCATAGCTTTAAGCATAGGAATGATTTTGGTTTTGATGATTTTTA
GTAAATTATTGCCTCATTATGAAAATAAAGATAAAAATATAAAAGAACATTTAATCGCTTTAAAAGTCTTAGAAAATAGC
TCTTTAATAGGCAAGAGCATAGAAGAGAACAAACTTAGAAATTTAGAGTTTTTATTTTTGGTAGAAATTCAAAGAAACAA
TCAAAGCATCACTCCCGTTTCACATAATGAAATCATAAATGCAAAAGATACACTTATATTTAGTGGAGATATTTCACAAC
TTGAAACTCTAAATAAATTTGATGGCTTAAAACTTGCAGATGGTTATGAGTTGAAAGAAAGTAAATTTATCGATGCAATC
ATTTCTCCAACTTCTAATTTAATTGGAAAAAGCGTAAAAGAAGCTAATTTTAGATCTAAATTTGACGCAGCTATTATTTC
TTTACAAAGAGGAGATGTGCATATCAAAAAAATTGGCGAAAGTGTTTTGCAAGCTGGAGATAGAATGATTTTAGCTGTGG
GTAAAGATTTTATCCTAAGAGATAATTTGGCTAAAAATTTTTATTTACTTTCAAATATCAAACAAAACGAAAAACTTGAC
ACTAAAAAAAGCTTATTGACCATAATGGCTTTTTTAAGTGTTATTGTTTTTTCGGCTTTAAATTGCATCTCTTTTACCAA
GGCTTTACTTATTTGTTTAGGCTTTTTACTACTTTGCAAATTTATCAAGCTTGATGAAATAAAAAGGCGTTTTCCTTTTG
ATATTTTTATCATAGTAGGTTCTTCTTTGGCAACTACTAAAGTACTTGTAGATAGCGGACTTGCCAAAGATTTAGCTCAA
CTTATCACAGGTTTTTTTGGACATTATGGAGTTTATGGAAGTTTTATAGGAGTTTATCTTTTAACCCTACTTTTAACCGA

ATTTATTACCAATAACGCTGCTGCGGCCTTAGCTTTCCCTATAGGTTTTGCCACAGCTCAAGCCTTGGGAGTAAATCCTT
TGCCCTTCATCTTTGCTGTAGCTTATGGTGCTAGTGCTGGGTTTATGATACCACATGGATACCAAACTCATTTAATGGTT
AGCTCGCTTTGTGATTACAAAATGACAGATTTTATAAAAATAGGTGCCATTGTTTCTATCGTGTATAGTGCTATAGTTCT
CATAGGCGTACCTTTAATCTTTAATTTTTAG

>HS19 Cluster X Campylobacter jejuni subsp. jejuni str. RM3420
TCACTCACCGATAAATTTTACCCCCATAATCTTACGCAACTCATCCATCAAAGACATCACTTTTAAAATCTCATCATGTT
TCATTGCATCGCATTCTATCTTACCTTGTTCTAAGGCCTCAAAACAAGCTTTAAATTCATACTCAAAACCATTGATTTGA
TTTGGCACAGCTAAGCTTTTGATACATTTTCTATCTTGATCGTAAAGCTCTATAAGAGTAGGGTTGTTGATATTATCCCC
ACGCTTATATAACCTTTTGTCCCATTGATAGTTCCTTTGCGATCATTTATCATAGCCACACTCGAATTTAAAAAAGCAAA
AGTTCCTTTACTAAACTCTAAAATCACCCCATTACTCTCATCTACACCGCTAGGCATTTTTTTACAAGTTGCCATTATAT
TTTTAATCTCATCTTGTATAAAACTAAGAGCAAAATTTAAAGTATAAACTCCCACATCAAGCAAAGCCCCACCCCAAGCT
CTAAGCTTTGTATGCGGTATATATCCTTAACATTATAAGCTAGATTTGCTTCTATACTTACTAACTCTCCTATAAAACTT
GCTCTATAAGTTCTTTAACCATGTTTATATAAGGCATATAACGCGTCCAAATACCTTCAGCCAAAAGCAAATTTTTAGCT
CTTGCTAGTTCTATAAGCTCTTTAGCCTCTTTGGAATTTAGTGTAAAAGACTTTTCACAAAGCACATTTTTAGCATTTTG
AAGGCAAAGTTTTATGTGAGAGTAATGTAAAGAATGCGGGGTATTGATATAAACAAGATCGATATTTTCATCTTTTACAA
GCTCTTCATAATTTGTATAAAATTTAGAAAAACCATAAGCACTAGCAAATTCCTTAGCATTACTTAAAGATCCTACCGCA
TAAGGAGTAAAACCGGCTTGTTTTAGCATGGGTGCGACTTTAGAAGCTATGGCTCCTGCACCTAAAAAAGCGACATTTCT
TCTCATTTATTTCCTTAAAATTTTTCTAAAATATGTAAATATTCTATAGTTTTATTTTGTTGACAAATACGCTTAGAATC
TGCTTTAAAACGCTGATAAATTTGGCTTTTAAAATCATATTTTCCATATTTTTCAAAGATTTGTCTAATTTGCTCTAAAG
AAAGCAAACCTTCATCGTTATAACTTAAAAAAATGAATTTAAAATCAGCATTTTTAATTAAATCTTCTAAAGCATCAAAA
ACCAAATTTTTCTTACACCAAGCAGATTTTTCATAGGCTCTTAAGCCCGTTTTTCCTTGTGGGATAAAATCATCATACAT
TGCAATGGAATTAAGCACATGATAATTTGCTCCATATTCTCTAGAATTGTAAGGCGGATCAAGATAAAGAATATCACCTT
TGATTTTTTTGATTAAATCATTTGCATTTTCATTAAAAACCAAGTGTTTTGTATGGGTGCATTCAAATTCTGCAGGCAAT
AATATAAGTTCTTTTTGTGCTGATGGTTTAAGATGTTTTAAAAAAGCCCCATAAACAGATGCTGTATTGGCTACTTTATC
AGCACTTTCCAACAAAGAGGCTAACAAAAAATAACATTCTTTTTCGTTAATAAAATTATCGTATTTCCACTCTGATATTT
TAATACGCATACTATCAATTTTCATTGCATTAAAATCGCTAAAATACTGCCTATTTTCGCCACCACCTAAAGAATAATGT
TTATATATTTTTCCTTTTATAGGATTAATTTTTGCAATATTATTTAATTCCTTAATTAATTCTTTGCTACGCTTTATTTT
TACAGTGTTTTCTATATAATTCTTAGCTAAAACAAAACTATAAAATTCTTTATCATTAGAAATCACTTTTTCAACTTTAT
TTTTAAATTTTTTCCCAACAGCTGAAGTTCCAGCAAATAAATCTATAAAAGTACATTGTTCTAAATTAATATCGCTTTTT
GCTAAAATATCATTAATACTCTCAAATAAAAAATCTAAAAGTTTTACTTTAGAGCCAATATAGTTCATTAAAATATTCTT
TCAAATTGAATTTGATTTTCAGATATAAAAAACTCTATATAAATCAAAGGTAAAGTATTATTATTAATACTAAATTTCTG
CACTTCTTCATAAAAAATTTTATAATTTGTAGAATTTTTATTAGTTTTTCTACATATAATTACCGGTAATATAGAGGATT
GATGATTAGGGACATAATATTGATTTAGCCAATTAATATATCTTTGCATTTGCAGTATTATTTCAGGATAAACTTCACAA
GATTTTAATTCTACGGGAATAACTTTTTTGTATATAAGTCCATTTATATTTATAGAGCTAGAAGCCATTATATCAATACT
TTGCATACCAACTCCACAACCTACTTCATTACCCAACCATTCTATATGCTCATTTGATAAAAGTCCAAAAAAATGTGTTT
TATAAAATTCTTGTAAAATATAAACCTGTAGATGCATTTCAAATTGTTTACCATCATAATATTTTTGAAATAATCTAGGC
AATATATTAATTTTTTCCATTCTGCCACTATAATTCAAACTAAAATTAGTATTTATTATTTTCTGTAAATATACATCAAA
ACTATATCTATTATAAGATAAATTAACAAAATTATTTTTTTTTCTTATTAAATTTATTAATCTATCACTTTCATACATAG
TAATCATAGTATTTCCACGATTACCTTTTAATTTTCTATAAATTAAACTCCATAGCATCTGATTTGGTGAAATAATATCT
TTAATTTCATCTAAAGCTTCCCATTCTGTAACACCTTCTGCATAAACTCCACAAGGTTCAATAAAACAACGAAAAGTTAA
AGATTTTTTCAACTCATCTTTTAAAAATTGTTTATCATCATTATTATCTAAAAACACCAATGAACTAATCTTAAAAAAAC
CATAAAATTTTCCTTCATAAATACCATTCTCGCAATTTTGCTGTAAATAAAAAATTACTAAATCACCAATTTGCACTCTT
GAAAAATCTGCAATTAAACATAAAATGGAATTTTCACTTGTTGGATGCAAAGATGTATTGTAGTTATTATTAAAGTCCAC
ATAATAGTCTTTATTTCCAGTGCCAACAAACCCATATTCTAAATGATATTTAAAAGTATTAACATCTACTATAAAAACAT
GAGTTGTCATATTATTTCAACTTTCTATGAATAGTATTTTTATCACATTTATTGCAAAATTTTATCTGACAAAACCTATT
AGGATTATTTGGATCACTTTCAAACTCATATCCGTCTTTTCTTAAATCTCTAATAATAGCAGCAGATTGAGAAGATAATT
TTCCAAAATCACAAATAGAACAATGCCATTCTTTATCTTTTACAATATCCAATATTTTTTCTTTATTATTCATATTTTAT
CCTTAAATTCATTTCATATTAAATTTATTTAATATAAACTCTTCTACTTCTTTGGAAATTCTTTTAATACTTTCTTTTCT
ATAAGCGACAGGATCATATTGATAACAACCGATACAGCCTAATTCTTTGGTATAATTTTCATCTCCTTTATAAAAATTAT
AAGGATTTCCTAAAATATTTCTAGCATCCCATCTTATATTTGTTTCTTTACATCTTTTACAAATTTGTCTTTTAATATCA
TTAGCGGCTTTACATAAAGGTTGAAAATCTTCTAATTTTTGCAAGTTAATATTAGATACTCTTAAATCTTGTTTTCTTCC
ATCTTTATGGTCGATTTCTATTTTCGTATTTTCAGATTTTCCATTAATTCCAAGCATAACACAATTTTTATTTTTATAAA
AATCTTTAATATCTTTCCTTATATTTTGATTAAATTGCGTTTGTGTATTAAAACCTGCGAGTCTTATTCTATCAATAGAA
TTTCCATTGCTCTTGGTCTTATCAAATTCTATCTTATATTTTTTTGCTAAAGCACTACTAGTACGACACCAACTTCCACC
ATTTCCAAGTTGTAAATTTTTGTATTTATTAACAAATTCTTTTACATCTACCCAACGAGAAAAACCGTTTTCATTCGGCA
AAGCAATTTCTAAAAATAACTCCATTTTAGTTACTTTCATTTTTGAATCCTTCAAATTTTATCCTATCAACAGAATTTCC
TTTTGTTATATTCGTATCTTTTGTTATTATATATGTTTTACTTAAATAACTGGTCTTTCTAGTAGATGGAAATCCATTTA
TCTGCTTTATTAATTTGTTCTCTAGTTATCCACTTGGAACAACCATTTTCATTTAAATTTGCTAATTTCAACAATTTTTT
TCATCTTCTGTCATTAAAACTCCTTTTTAAATACAAAAATATACTCATGCTTAAAAATATAATAATCACTCCTTAATGCT
CTATATCTCCATATAGCATTAATACCTAGTTTTCCACGATTTCCTTCTATATTTTTAATAACAATTCCCTTTAGTTTAAC
TTTAAAATTTTGTTTTATTAAATTCATAAGCTCAAAACCCAAAGGAACAATTTCACTATTTCTATATAAATCTGCTATCA
CTAAAGCAAAATAACGATTTTTTTCTAAGTATTTTAACAAATTTTGACAAGTAAGTAAAATTTTATCTTTAAATTCTTTC
AAATCTGAAATTTGACTTAAATCCTCTTTTTTCTCACTAAATTTAACTATATCCATATAAGGTGGATGTAAAATAATAAA
CTGAGATTTTTTACTTTCAAGTTTATTCAAAGCATTTTCTAACAACTCATCAACTTTTTTTATATCAGTATTATCACAAT
TTCCTAAAAAATAGCGCTTATTATCAAAATTTATCCCAAAGTTGTCTAGTACAAATTCCAAAATGCTCTCATTAATATCA
AATCCTATGCATTTTCTATTTAATTTTTCACATTCATAAAGCGTTGTTCCACTTCCTAAAAAAGGATCTATAATTAAATC
ATCTTTTTTTGTATAACGACGGATGAGCTGATTAGGAATTTGTGGTATAAAATTACCATGATAAATATTTCTATGCTTTC
CACTTTTATCACGACTAGCAATAAGCCATAAAGAATCTGTATTTATATCACAACTTTTCCAGTTGTTCAAATCAAGATCG
TTAAATTTCAC

>LOSA Campylobacter jejuni subsp. jejuni str. RM3420
AATGATGAATATCCTTTTATGCCAAGGCAAAAATTGCCTAAAACATATATGAGCAATGGTGCAATTTATATTTTAAAGAT
AAAAGAATTTTTAAACAATCCTAGCTTTTTACAAAGCAAAACCAAGCATTTTTTAATGGATGAAAGCTCAAGTTTAGATA
TTGACTGTTTGGAGGATTTAAAAAAGGCTGAACAGATATGGAAAAAATAACCTTAAAATGCAATAAAAATATATTAAATT
TATTAAAGCAATATAATATTTATACAAAAACTTATATAGAAAATCCTAGAAGATTTTCAAGACTAAAAACCAAAGATTTT
ATAACCTTTCCATTGGAAAACAATCAACTAGAGAGTGTAGCGGGGCTGGGGATAGAAGAATATTGTGCTTTTAAATTTAG
CAATATCTTACATGAAATGGGTTCATTTTCTTTTAGCGGATCTTTTCTACCTCATTATACAAAAGTTGGAAGGTATTGTT

>LOSA1 Campylobacter jejuni subsp. jejuni str. RM3420
CAACAAAACAAAGTTTTTCTTCAAATATACACTCTTTAGAGCAATTAAAGTATAAACACAGGATATTATTTCACACTGAA
TTAAATAATTATCATTTTCCTTTTTTAAAAAAACATAGAGCTCAAGATATTTATAAATATAATTGGATAAGTATTGAAGA
ATTTAAAAAAATTCTATTTACAAAATATTAATCATAAAATAGAACCTTCTATGATTTCAAAAGAAACTCTAAAAAAAATAT
TCTTAACATTGTTTTAAAAATTTTTTATATTTAAATAAAATTTTTAAAGTTAAAATATTTATTTTAGCTAACAATGTAAC
CATTAATTTTGTTCTTTTTATTTTATATATTTGAATATATAGCAAATATTTAATTAGCACATAGAGAACGCTACAATACT
TGTTTAAAATATAATTTTGCCTTAAATAGTTTAAAACCAACTGCAACTCTTGAATATTATTTTTAACAAGCACTTCATTC
TTAGTATTACAAATTGAATT

>HS19 CPS marker Campylobacter jejuni subsp. jejuni str. RM3420
GGCAACAAACAAACATATTCAGAAATTTTTCCTTCATAGAATTTCATATCGCTATATTTTGGTGGATTAAAATTAAAATA
TTCAGAGATTAGTTTAATACTTTCAAAACATTTGTTGCCCAAAATTGCATTTGTATCTAAAAAATAAATTTTATCTTGTT
TTAAAAATTTCCATAATAAAGAATCATGAAAACACATAAATTTGTTATCAATAAATGCTTCTGAAATATCAACACAGGGA
AAATGTGAATTAAATCCAATATTAGCATATCCAATCCTGTTGCTCATAACGCATTCAATATTAGTGCCAAAATCTACAAT
ATTTAAATAATTAACACCATGTCTTTTACCTCCAACATGAGATCTTAAAGAAGAAATAGGATCCCTTACAAGACAAATAC
TTCTACATGGTGGAATAAGAGAGTAATACTTTGAGGCATTTTCATCCTCG
